# Prevalence and trends of advanced HIV disease among antiretroviral therapy-naïve and antiretroviral therapy-experienced patients in South Africa between 2010-2021: a systematic review and meta-analysis

**DOI:** 10.1186/s12879-023-08521-4

**Published:** 2023-08-22

**Authors:** Marcel K. Kitenge, Geoffrey Fatti, Ingrid Eshun-Wilson, Omololu Aluko, Peter Nyasulu

**Affiliations:** 1 Division of Epidemiology and Biostatistics, Department of Global Health, Faculty of Medicine and Health Sciences, Stellenbosch University, Cape Town, South Africa; 2 Tuberculosis and HIV investigative Network (THINK), Durban, Kwazulu-Natal, South Africa; 3 Kheth’Impilo AIDS Free Living, Cape Town, South Africa; 4 Division of Infectious Diseases, School of Medicine, Washington University in St. Louis, St. Louis, MO USA; 5 Faculty of Health Sciences, School of Medical Sciences, Department of Biostatistics, University of the Free State, Bloemfontein, South Africa; 6 Division of Epidemiology and Biostatistics, School of Public Health, Faculty of Health Sciences, University of the Witwatersrand, Johannesburg, South Africa

**Keywords:** Burden, Advanced HIV disease, ART-naïve, ART-experienced, South Africa

## Abstract

**Background:**

Despite the significant progress made in South Africa in getting millions of individuals living with HIV into care, many patients still present or re-enter care with Advanced HIV Disease (AHD). We aimed to estimate the prevalence of AHD among ART-naive and ART-experienced patients in South Africa using studies published between January 2010 and May 2022.

**Methods:**

We searched for relevant data on PubMed, CINAHL, Scopus and other sources, with a geographical filters limited to South Africa, up to May 31, 2022. Two reviewers conducted all screening, eligibility assessment, data extraction, and critical appraisal. We synthesized the data using the inverse-variance heterogeneity model and Freeman-Tukey transformation. We assessed heterogeneity using the I^2^ statistic and publication bias using the Egger and Begg’s test.

**Results:**

We identified 2,496 records, of which 53 met the eligibility criteria, involving 11,545,460 individuals. The pooled prevalence of AHD among ART-naive and ART-experienced patients was 43.45% (95% CI 40.1–46.8%, n = 53 studies) and 58.6% (95% CI 55.7 to 61.5%, n = 2) respectively. The time trend analysis showed a decline of 2% in the prevalence of AHD among ART-naive patients per year. However, given the high heterogeneity between studies, the pooled prevalence should be interpreted with caution.

**Conclusion:**

Despite HIV’s evolution to a chronic disease, our findings show that the burden of AHD remains high among both ART-naive and ART-experienced patients in South Africa. This emphasizes the importance of regular measurement of CD4 cell count as an essential component of HIV care. In addition, providing innovative adherence support and interventions to retain ART patients in effective care is a crucial priority for those on ART.

**Supplementary Information:**

The online version contains supplementary material available at 10.1186/s12879-023-08521-4.

## Background

Efforts to quickly expand access to human immunodeficiency virus (HIV) care and treatment have led to a significant number of individuals starting antiretroviral therapy (ART). The most notable increase has occurred in South Africa, which is home to 7.5 million of the estimated 37.7 million people living with HIV (PLHIV) [1]. Access to ART in South Africa has grown from 103,300 to 2005 to 5,500,000 in 2021. Despite significant progress in expanding access to ART, the burden of Advanced HIV Disease (AHD) in South Africa remains high [1–4]. AHD is defined as a CD4 count of fewer than 200 cells/mm^3^ or a WHO stage 3 or 4 event in adults and adolescents, and all children with HIV younger than five years old are considered to have AHD [5, 6]. The proportion of people presenting with AHD has remained relatively unchanged in the past five years, despite the number of PLHIV receiving ART in low- and middle-income countries (LMIC) more than doubling over this period [4, 7, 8]. These patients are at high risk of death mainly due to Tuberculosis and cryptococcal meningitis, even after starting ART (which can increase the inflammatory response) and the risk increases with decreasing CD4 cell count [9].

AHD is prevalent in Sub-Saharan Africa (SSA), with studies reporting between 32% and 71% of patients initiating care with AHD, and up to 60% of patients presenting for care with AHD after disengagement [10, 11]. A large multi-country cohort study using data from HIV treatment programs in 6 SSA countries during 2005–2018 showed that South Africa had the highest percentage of adults with AHD (59.7%) starting ART. However, the study also noted that the percentage of adults starting ART with AHD steadily declined over time [4].

Contrary to the expectation that PLHIV will progress from testing and early ART initiation to consistent drug adherence and viral suppression, many PLHIV instead need assistance re-engaging in care with AHD following treatment failure or interruption [12]. This is a concerning trend observed in countries with established HIV programs, where an increasing number of patients present for care with AHD after a period of treatment interruption or ART-experience [13]. In a study by Osler et al. [2] in South Africa, the proportion of ART-experienced patients with a CD4 count less than 50 cells/mm^3^ increased from 14.3% to 2008 to 56.7% in 2017 and this group seeks hospital care following ART interruption or virological failure.

Previous meta-analysis studies have primarily focused on analysing temporal trends of CD4 count at presentation to care and treatment initiation, globally [14], in developed countries [15] and in low and middle-income countries [16]. It is crucial to estimate the prevalence of AHD among ART-naive and ART-experienced patients to understand the outcomes of ART programs, guide HIV prevention efforts, and predict the need for adjunctive therapies [17–19]. The true estimates of AHD among ART-naïve and ART-experienced patients in South Africa are not well known. This systematic review and meta-analysis aimed to estimate the prevalence of AHD among ART-naive and ART-experienced patients in South Africa, using data from studies published between January 1, 2010 and May 31, 2022. Furthermore, we sought to examine trends in the prevalence of AHD throughout this period.

## Methods

This study employed a systematic review and meta-analysis design and the reporting of this systematic review was guided by the standards of the Preferred Reporting Items for Systematic Reviews and Meta-analyses (PRISMA) Statement [20]. The protocol for this systematic review was registered and published on the International Prospective Register of Systematic Reviews (PROSPERO) with the registration number: CRD42021245429.

### Criteria for considering studies for review

#### Type of studies

We included both published and unpublished observational studies (such as cross-sectional and cohort studies), as well as HIV program data. Additionally, we included population-based cross-sectional studies or surveys. Studies had to be published between January 1, 2010 and May 31, 2022 to be eligible. However, we excluded experimental studies, case studies, case series, duplicate reports, commentaries, and reviews from our analysis.

#### Condition

Published and unpublished needs Number of patients with AHD., We defined AHD as any CD4 < 200 cells/mm^3^ or a WHO stage 3 or 4 events. All children with HIV younger than five years old should be considered as having AHD. We calculated the prevalence by dividing the number of observed AHD by the total number of observed PLHIV on ART and expressed it as a percentage.

#### Context

We included studies conducted in South Africa between January 1, 2010 and May 31, 2022.

#### Population

ART-naïve and ART-experienced (all age groups) patients were considered for this review.

### Search strategy and data source

We conducted a comprehensive search of studies in various databases including PubMed, CINAHL, Scopus, Scielo, and Africa Wide, with a geographical limited to South Africa. A hand search of citations from selected studies was conducted to identify additional studies missing from the original electronic searches and sought input from experts for any additional studies. Conference proceedings of the Conference on Retrovirus and Opportunistic Infections (CROI) and International Aids Society Conference (IAS) were screened to retrieve information from any further study that may not have been included in the electronic databases. Our search was conducted on July 16, 2021 and updated on May 31, 2022, and we limited the search to studies published between January 1, 2010 and May 31, 2022 and no language restriction was applied. The specific electronic search strategy is outlined in supplementary appendix 1.

### Screening of studies for inclusion

The records of identified studies from the search databases were imported into COVIDENCE [https://app.covidence.org/], a software program used for managing systematic reviews. We removed duplicate abstracts before screening. Two reviewers, Marcel Kitenge (MK) and Omololu Aluko (OA), screened studies for initial inclusion using titles and abstracts. Both MK and OA independently evaluated the full text of studies that met the criteria for full-text screening, using a predefined eligibility checklist. Any disagreements about study eligibility were resolved through discussion and consensus.

### Data extraction

After identifying eligible studies, two reviewers (MK and OA) independently extracted data from each study using a standardised pre-piloted data extraction form in COVIDENCE. We also extracted information on the characteristics of included studies, such as authors, citations, year of publication, study period, provinces, populations (whether inpatient or outpatient), study design used, and median CD4 count at enrolment. In case of missing information, we clarified the conducted study or the studies that had relevant data, which were not reported in the published manuscript, we contacted the authors for additional information.

### Assessment of study quality and risk of bias

We evaluated the methodological quality of the included studies by assessing their internal validity, and generalizability of results. A ten-item rating system developed by Hoy et al. [21] was used to evaluate aspects such as sampling, sample size, outcome measurement, outcome assessment, and statistical reporting. Each item was assigned a score of 1 (yes) or 0 (no), and the scores were summed to generate an overall quality score ranging from 0 to 10. Each study was classified as having a low, moderate, or high risk of bias based on the number of questions answered as “yes”. Studies with scores higher than 8 were classified as low risk, 6–8 as moderate risk, and 5 or lower as high risk. Two reviewers (MK and OA) independently assessed study quality, and any discrepancies in rating were resolved through discussions and consensus. Eligibility assessment, data extraction, and assessment of the risk of bias were performed using COVIDENCE.

### Data analysis

We pooled data on the prevalence of AHD using STATA version 17 [22] and the metaprop package [23]. We employed a random-effects meta-analysis framework as we anticipated variability in the prevalence estimates from different studies. The package models the prevalence estimates using the exact binomial distribution and then applies the Freeman-Turkey double arcsine variance stabilizing transformations, normalizing the estimates before pooling and then back-transforming the estimates. The pooled estimates were then computed using the method described by DeSimonian and Kacker [24].

We assessed heterogeneity between studies using Cochran’s Q statistic [25] (expressed as X^2^ and p-values) and the I^2^ statistic. We also explored sources of heterogeneity through subgroup analyses and meta-regression analysis. The following study characteristics were evaluated using univariate meta-regression models: study design (cross-sectional or non-cross-sectional), sample size (continuous), median CD4 count, methodology quality score, and publication year. Given included studies had substantial heterogeneity, we conducted a sensitivity analysis to investigate if the results were influenced by a single study. Due to high degree of heterogeneity, we presented pooled estimates with corresponding prediction intervals as prediction intervals are a more conservative way to incorporate uncertainty in the analysis.

We also estimated trend or change in prevalence of AHD among ART-naive patients overtime by fitting meta-regression model, modelling it as a weighted linear function of calendar year. We also assessed the presence of publication bias by examining funnel plots, and formally using statistical testing such as the Egger test [26] and the Begg’s test [27]. A p-value < 0.1 was considered as indicating significant publication bias.

## Results

### Search results

**Fig. 1:**
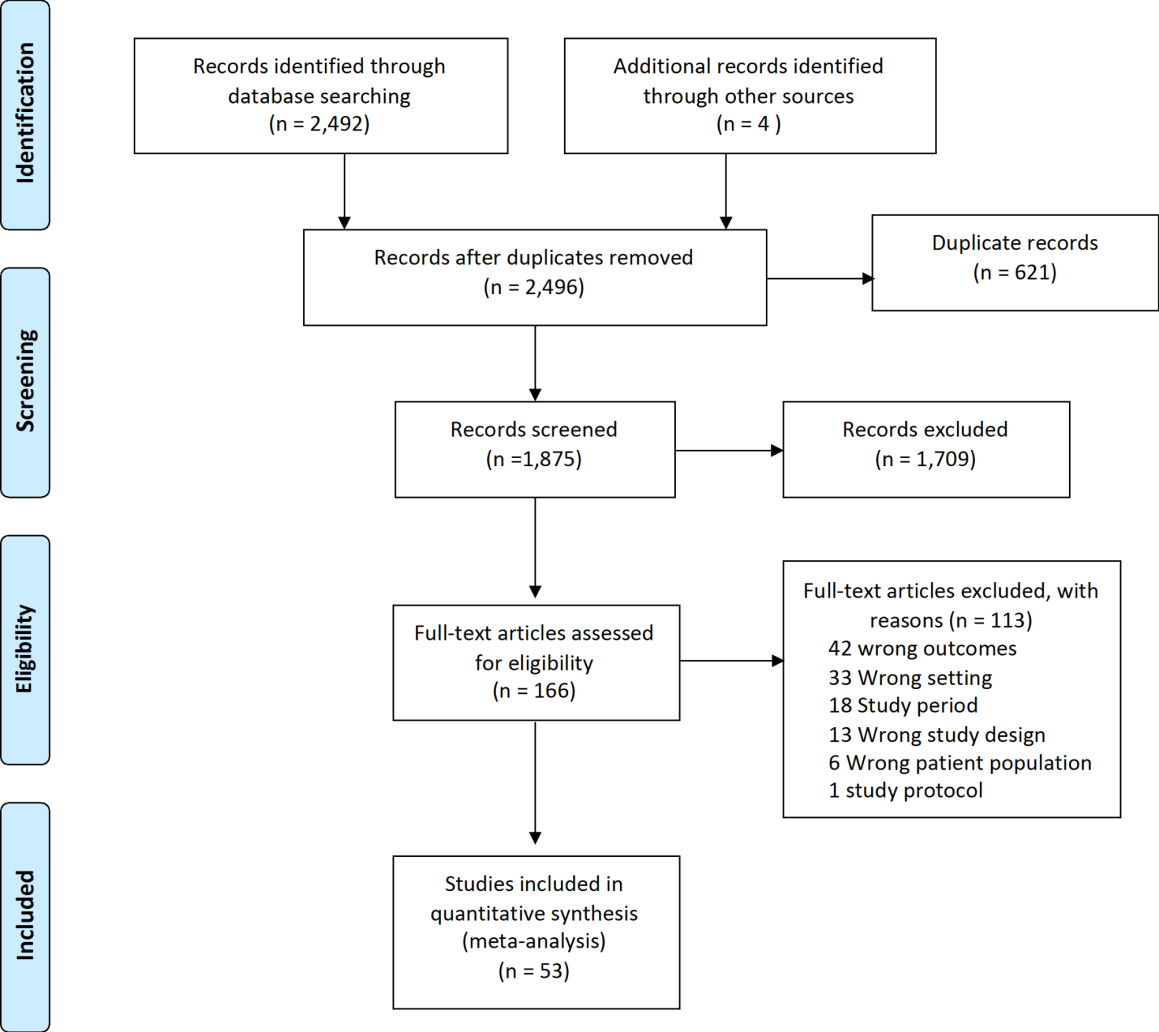
PRISMA flowchart showing search, selection of and final included studies

We identified 2,496 records from searches. After removing duplicate and excluding 1,709 irrelevant records, the full texts of 166 studies were assessed for eligibility; 53 of these were included in this systematic review (Fig. 1). The reasons for exclusion are listed in Fig. 1.

### Characteristics of included studies

The 53 studies published between January 2010 to January 2022 consisted of a total of 11,545,460 individuals who were HIV positive. Twenty-four of the studies were cross-sectional [13, 28–50]; two were cross-sectional population surveys [51, 52], thirteen were longitudinal analyses using routine electronic HIV programmatic data [2–4, 53–62]; ten were prospective cohort studies [63–72] and the remaining 4 were retrospective cohort studies [73–76]. The study population for 48 studies was among adults: two studies enrolled adolescents [50, 62] and three enrolled children [30, 38, 39].

Five studies enrolled inpatients while the remaining enrolled outpatients. We provided detailed information about the included studies and summarised key features in Table 1.

**Table 1:** Characteristics of included studies

Study authors	Year	Province	Study design	Study period	Total sample	Number of AHD	Assessment method	Population	ART status*	Median CD4	Setting	ART-experienced Total sample	Number of AHD (ART-experienced)	Summary score	Risk of bias	Refence
Adeniyi	2018	Eastern Cape	Cohort (P)**	2015–2016	594	307	CD4	Adults	Naive		Outpatients			8	Moderate	[69]
Bock	2018	Western Cape	Cohort (P)**	2014–2015	2423	631	CD4	Adults	Naive	328	Outpatients			8	Moderate	[70]
Boulle	2014	National	Cohort (R)**	2005–2010	30,467	24,193	CD4	Adults	Naive	102	Outpatients			7	Moderate	[54]
Carmona	2018	National	Cohort (R)**	2005–2016	8,036,919	2,973,032	CD4	Adults	Naive		Outpatients			7	Moderate	[3]
Cassidy	2022	Western Cape	Cohort (R)**	2008–2018	5738	1349	CD4	Adolescents	Naive	318	Outpatients			9	Low	[62]
Chihana	2019	KwaZulu-Natal	Cross-sectional	2013	1400	130	CD4	Adults	Both		Outpatients			10	Low	[52]
Cholera	2017	Gauteng	Cross-sectional	2013	301	159	CD4	Adults	Naive	188	Outpatients			10	Low	[41]
Clouse	2013	Gauteng	Cohort (P)	2010	1430	743	CD4	Adults	Naive	268	Outpatients			10	Low	[65]
Conan	2019	KwaZulu-Natal	Cross-sectional	2018	834	38	CD4	Adults	Naive	483	Outpatients			10	Low	[51]
Dorward	2020	KwaZulu-Natal	Cross-sectional	2017	390	93	CD4	Adults	Naive	366	Outpatients			7	Moderate	[48]
DrainPaul	2013	KwaZulu-Natal	Cross-sectional	2011	830	279	CD4	Adults	Naive	186	Outpatients			8	Moderate	[36]
Dramowski	2011	Gauteng	Cross-sectional	2007	510	408	WHO stage	Children	Both	700	Inpatients			5	High	[30]
Feucht	2016	KwaZulu-Natal	Cross-sectional	2010	250	205	WHO stage	Children	Naive	822	Inpatients			8	Moderate	[39]
Fomundam	2017	Gauteng	Cross-sectional	2015	12,413	3973	CD4	Adults	Naive		Outpatients			8	Moderate	[43]
Glencross	2020	Limpopo	Cohort (R)**	2005–2015	57,490	19,854	CD4	Adults	Naive	283	Outpatients			8	Moderate	[61]
Haas	2020	Western Cape	Cohort (R)**	2004–2017	58,664	24,693	CD4	Adults	Naive	186	Outpatients			10	Low	[60]
Haddow	2012	KwaZulu-Natal	Cross-sectional	2006	498	343	WHO stage	Adults	Naive	106	Outpatients			8	Moderate	[34]
Hunt	2017	KwaZulu-Natal	Cross-sectional	2013	1299	687	CD4	Adults	Naive	230	Outpatients			7	Moderate	[40]
Kamkuemah	2015	Western Cape	Cohort (R)**	2006	1091	621	CD4	Adults	Naive	154	Outpatients			8	Moderate	[55]
Kranzer	2012	Western Cape	Cross-sectional	2010	164	20	CD4	Adults	Naive	385	Outpatients			7	Moderate	[33]
Larsen	2019	Gauteng	Cross-sectional	2015	6826	2594	CD4	Adults	Naive		Outpatients			10	Low	[45]
Larson	2010	Gauteng	Cohort (R)**	2009	9399	3293	WHO stage	Adults	Naive	73	Outpatients			7	Moderate	[53]
Lawn	2011	Western Cape	Cohort (P)	2011	235	125	CD4	Adults	Naive	171	Outpatients			10	Low	[64]
Lewis	2012	KwaZulu-Natal	Cohort (R)**	2016–2018	2575	210	CD4	Adults	Naive	106	Outpatients			10	Low	[76]
Lilian	2019	Gauteng	Cohort (R)**	2017–2018	32,290	3737	WHO stage	Adults	Naive		Outpatients			10	Low	[58]
Lurie	2014	Western Cape	Cohort (P)	2009–2011	1465	991	CD4	Adults	Naive		Outpatients			10	Low	[68]
Maduna	2015	National	Cohort (R)**	2008	5976	3431	CD4	Adults	Naive	207	Outpatients			7	Moderate	[75]
Magidson	2019	Western Cape	Cohort (P)	2010	190	65	CD4	Adults	Naive	418	Outpatients			8	Moderate	[72]
Manicklal	2014	Western Cape	Cross-sectional	2012	748	126	CD4	Adults	Naive		Outpatients			5	High	[37]
Maskew	2011	Western Cape	Cross-sectional	2008–2009	404	309	CD4	Adults	Naive	100	Outpatients			8	Moderate	[29]
Meintjes	2015	Western Cape	Cross-sectional	2012–2013	209	137	CD4	Adults	Both	134	Inpatients	376	234	8	Moderate	[13]
Mnyani	2017	Gauteng	Cross-sectional	2005–2015	198	142	CD4	Adults	Naive	236	Inpatients			8	Moderate	[42]
Naidoo	2014	KwaZulu-Natal	Cohort (R)**	2007–2010	969	722	CD4	Adults	Naive	128	Outpatients			10	Low	[74]
Ndlovu	2014	Gauteng	Cohort (P)	2004–2010	2489	1322	WHO stage	Adults	Naive	83	Outpatients			8	Moderate	[67]
Nglazi	2012	Western Cape	Cohort (R)**	2008–2010	893	102	CD4	Adults	Naive	382	Outpatients			7	Moderate	[73]
Nir	2021	Western Cape	Cross-sectional	2014–2019	181	95	CD4	Adults	Naive	225	Outpatients			9	Low	[49]
Nyakato	2022	National	Cross-sectional	2004–2017	2733	472	CD4	Adolescents	Naive		Outpatients			10	Low	[50]
Oni	2011	Western Cape	Cross-sectional	2008–2010	228	45	CD4	Adults	Naive	312	Outpatients			10	Low	[31]
Osler	2018	Western Cape	Cohort (R)**	2008–2017	1,771,201	397,214	CD4	Adults	Both		Outpatients			10	Low	[2]
Otwombe	2013	Gauteng	Cohort (P)	2006–2009	891	616	WHO stage	Adults	Naive	67	Outpatients			10	Low	[66]
Patel	2010	KwaZulu-Natal	Cross-sectional	2009	81	62	CD4	Adults	Naive	132	Outpatients			7	Moderate	[28]
Patten	2020	National	Cohort (R)**	2005–2018	283,134	158,825	CD4	Adults	Naive	140	Outpatients			8	Moderate	[59]
Peter	2012	Western Cape	Cross-sectional	2009–2010	281	78	CD4	Adults	Naive	86	Inpatients			8	Moderate	[35]
Rane	2018	KwaZulu-Natal	Cohort (P)	2013–2016	1271	372	CD4	Adults	Naive	335	Outpatients			8	Moderate	[71]
Rossouw	2015	KwaZulu-Natal	Cross-sectional	2008–2012	65	54	WHO stage	Children	Naive	558	Outpatients			9	Low	[38]
Shigayeva	2019	KwaZulu-Natal	Cohort (R)**	2008–2018	27,929	10,125	CD4	Adults	Both		Outpatients	726	411	10	Low	[57]
Sogbanmu	2019	Eastern Cape	Cross-sectional	2016–2017	335	153	WHO stage	Adults	Naive	350	Outpatients			10	Low	[46]
Tendesayi	2016	National	Cohort (R)**	2010–2014	1,070,900	498,785	CD4	Adults	Naive	213	Outpatients			10	Low	[56]
Theron	2011	Western Cape	Cross-sectional	2007–2010	123	66	CD4	Adults	Naive	182	Outpatients			10	Low	[32]
VanRie	2011	Gauteng	Cohort (P)	2004–2007	7536	6073	CD4	Adults	Naive	99	Outpatients			7	Moderate	[63]
Venter	2010	Gauteng	Cross-sectional	2017	553	266	CD4	Adults	Naive	199	Outpatients			5	High	[44]
Zaniewski	2020	National	Cohort (R)**	2005–2018	94,167	56,228	CD4	Adults	Naive		Outpatients			8	Moderate	[4]
VanSchalkwyk	2020	Gauteng	Cross-sectional	2015–2016	5280	637	CD4	Adults	Naive	32	Outpatients			8	Moderate	[47]

Footnotes: *ART status = ART naïve, ART experienced, or both; **Cohort (P) = prospective; Cohort (R) = retrospective analysis of HIV program data,

### Risk of bias assessment of included studies

In the assessment of the methodological quality, three studies were deemed to be of poor methodological quality. Of the remaining 50 records, 28 studies were deemed to be of moderate quality and 22 studies of high methodological quality (supplementary, appendix 2).

### Prevalence of AHD among ART-naïve patients in South Africa

**Fig. 2:**
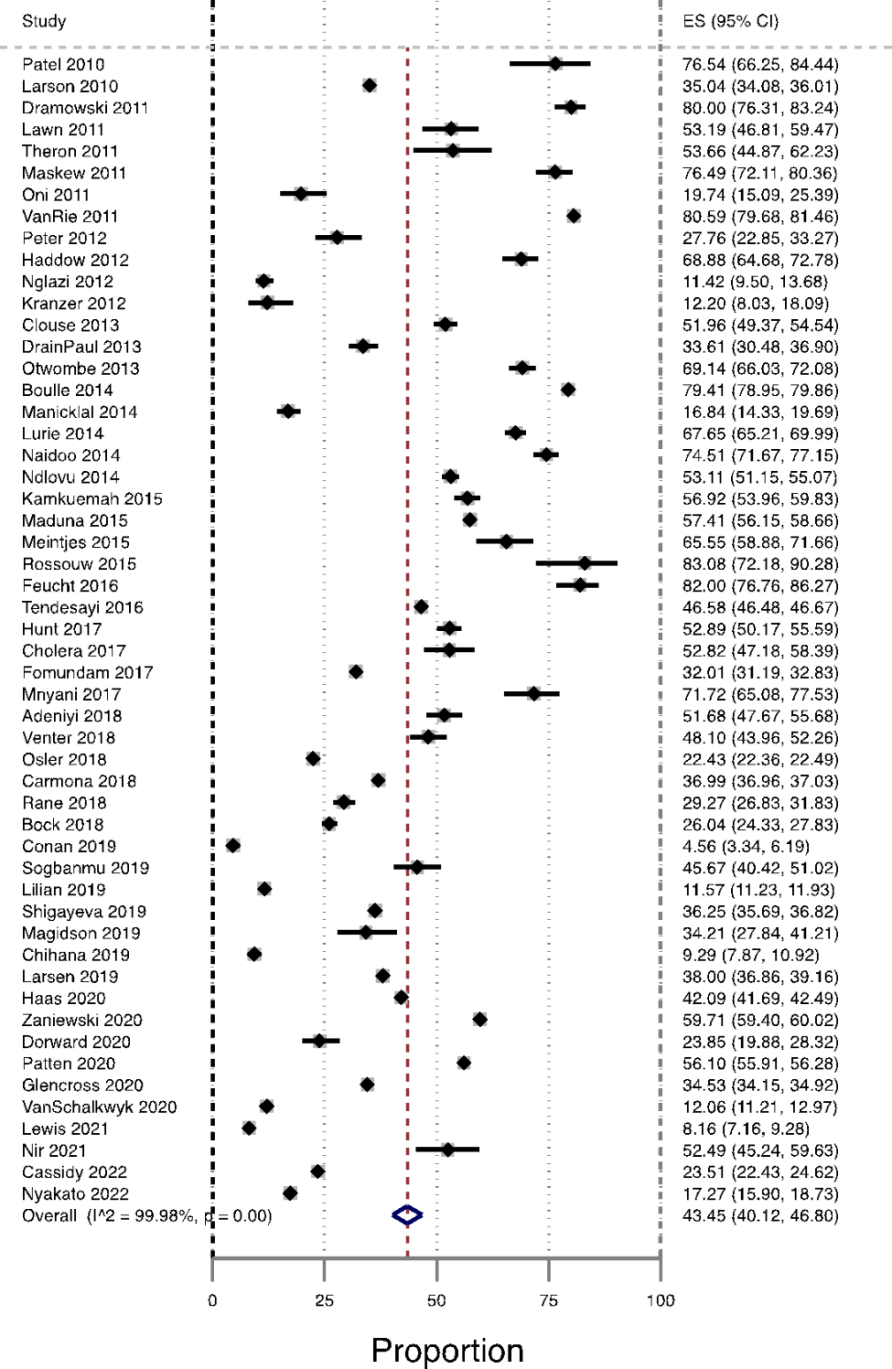
Forest plot of prevalence of AHD among ART-naïve in South Africa, from studies published from 2010–2022

The 53 studies had a combined total of 11,545,460 PLHIV with 4,204,835 patients having AHD at ART enrolment. The prevalence of AHD in South Africa ranged between 5 and 82%. The pooled overall prevalence of AHD among ART-naïve patients was 43.4% (95% CI 40.1–46.8%, n = 53 studies). There was substantial heterogeneity among included studies (I^2^ = 99.9, P < 0.001) as shown in Fig. 2. Our estimates in prediction intervals analysis showed that the prevalence of AHD among ART-naïve patients was 44.3% (95% CI 38.0 to 50.0).

### Prevalence of AHD among ART-naïve patients by age group

**Fig. 3:**
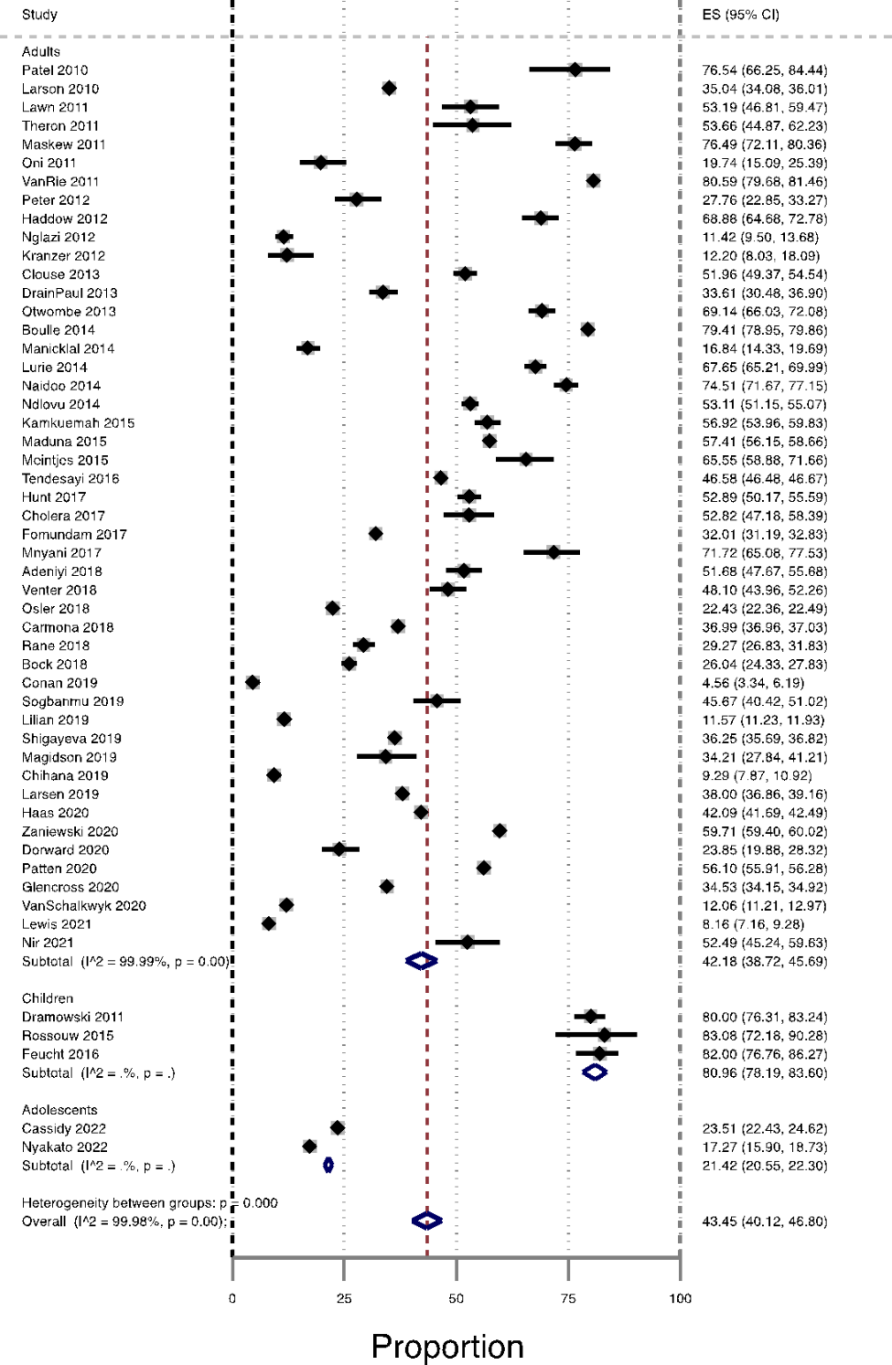
Prevalence of AHD among ART-naïve patients in South Africa by age group, from studies published during the period 2010–2022

The lowest pooled prevalence of AHD among ART-naïve was observed among adolescents 21% (95% CI 20.55–22.30%, n = 2 studies) and adults’ group 42.2% (95% CI 38.7–45.7%, n = 48 studies). The highest pooled prevalence of AHD among ART-naïve was observed among children 80.9% (95% CI 78.2–83.6%, n = 3 studies). The test for subgroups differences indicated that there was a statistically significant subgroup effect for age (P < 0.001), Fig. 3.

### Prevalence of AHD among ART-native patients by assessment methods: CD4 versus WHO clinical staging system

**Fig. 4:**
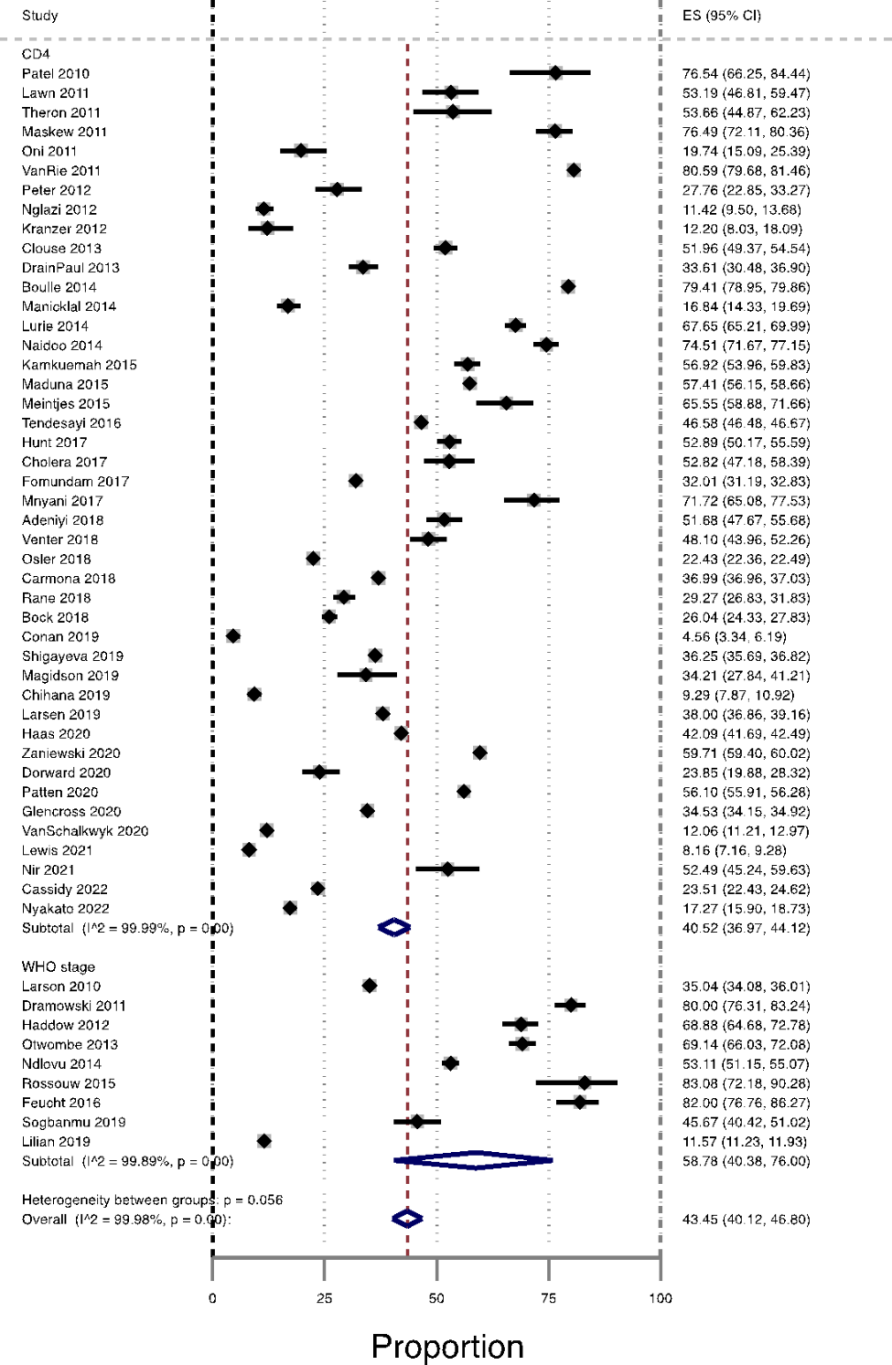
Prevalence of AHD among ART-naïve patients in South Africa by Assessment methods (CD4 count and WHO clinical staging system), from studies published during the period 2010–2022

The pooled prevalence of ADH among ART-naïve patients was significantly higher among studies that used the WHO clinical staging system to assess AHD, 58.8% (95% CI 58.8–76.0%, n = 9), compared with a pooled prevalence of AHD estimate in 44 studies that used CD4 count criteria; 40.5% (95% CI 37.0% to 44.1, n = 44), Fig. 4.

Additionally, the prevalence of AHD among ART-naïve patients was 66.1% (95% CI 45.2–84.2%, n = 5) in studies conducted in hospital settings (inpatients) compared with 41.1% (95% CI 37.7–44.6%, n = 48) in studies conducted in ambulatory studies or outpatients (Appendix 3, Fig. 1) p < 0.001. We further conducted a subgroup analysis evaluating the prevalence of AHD among ART-naïve patients across study designs, the highest prevalence of AHD was observed among prospective cohort studies with 51.8% (95% CI 36.7–66.8%, n = 10), followed by cross sectional studies, retrospective analysis of HIV program data, retrospective cohort studies and cross-sectional survey with 46.9%, 41.1%, 35.1% and 7.3%, respectively, (Appendix 3, Fig. 2). Moreover, from sub-analyses, we observed that ADH among ART-naïve patients was more common among studies analysing national HIV cohort, followed by studies conducted in Eastern Cape province, Gauteng, KwaZulu-Natal, Western Cape, and Limpopo (Appendix 3, Fig. 3).

### Sensitivity analysis

We performed one study leave out at a time sensitivity analysis and its result showed that the polled estimated prevalence of AHD among ART-naïve patients obtained when each of the included studies was left out from the analysis at a time was within the 95% confidence limit of the pooled estimate of AHD when all studies were pooled together. The average prevalence of AHD among ART-naïve patients when one of the 53 studies was left out from the analysis ranges between 44.0% (95% CI 38.0–50.0%) and 45.0% (95% CI 39.0–51.0%) (Appendix 3, Fig. 4).

### Prevalence of AHD among ART-experienced patients

**Fig. 5:**
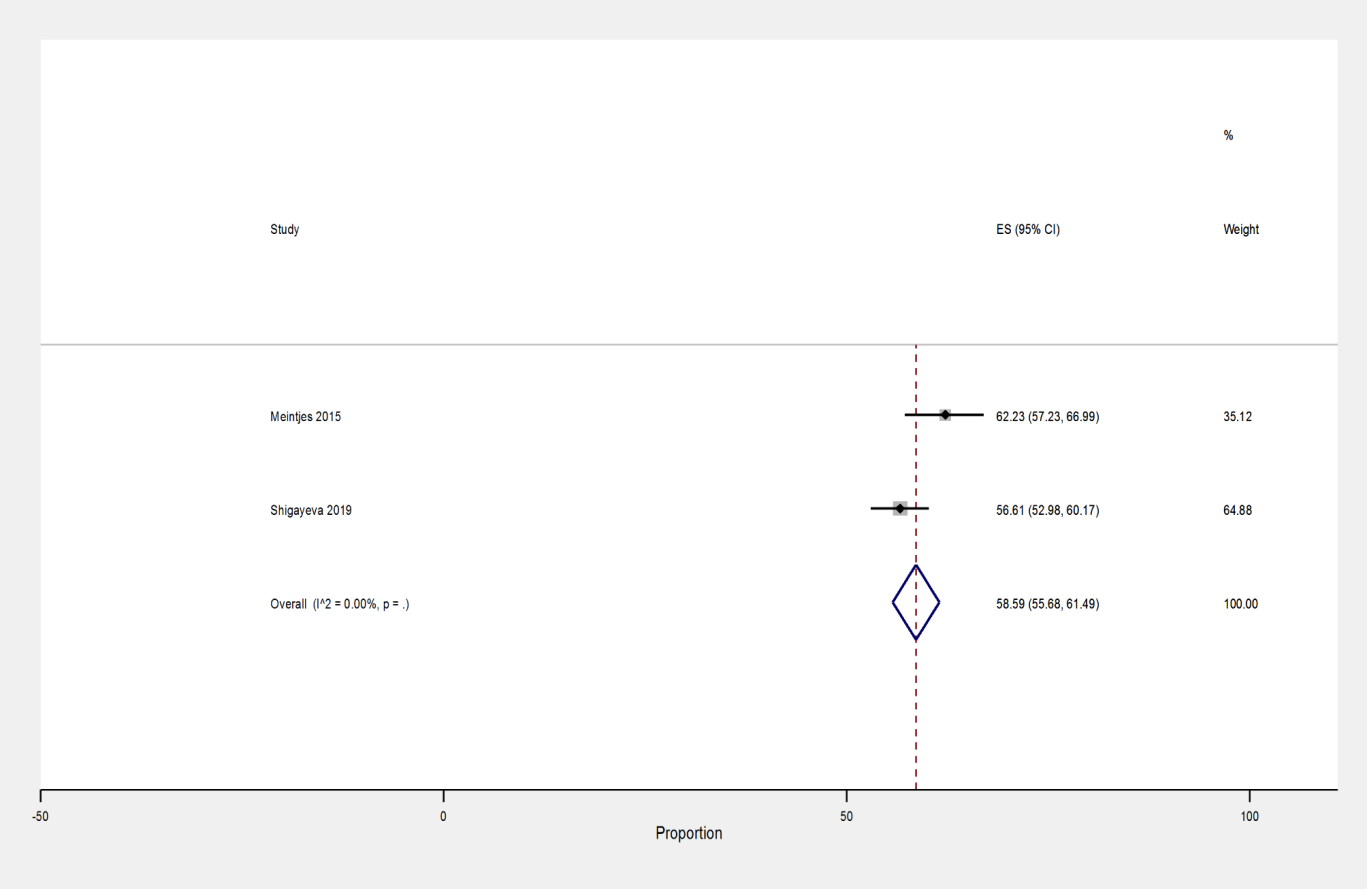
Prevalence of AHD among ART-experienced patients in South Africa, from studies published during the period 2010–2022

We identified two studies assessing the prevalence of AHD among ART-experienced patients, including one cross-sectional and a retrospective analysis of HIV programmatic data which were included in the pairwise meta-analysis. The pooled prevalence of AHD among ART-experienced patients provided by two studies with 1,102 participants was 58.6% (95% CI 55.7 to 61.5%, n = 2), Fig. 5.

### Temporal trend

**Fig. 6:**
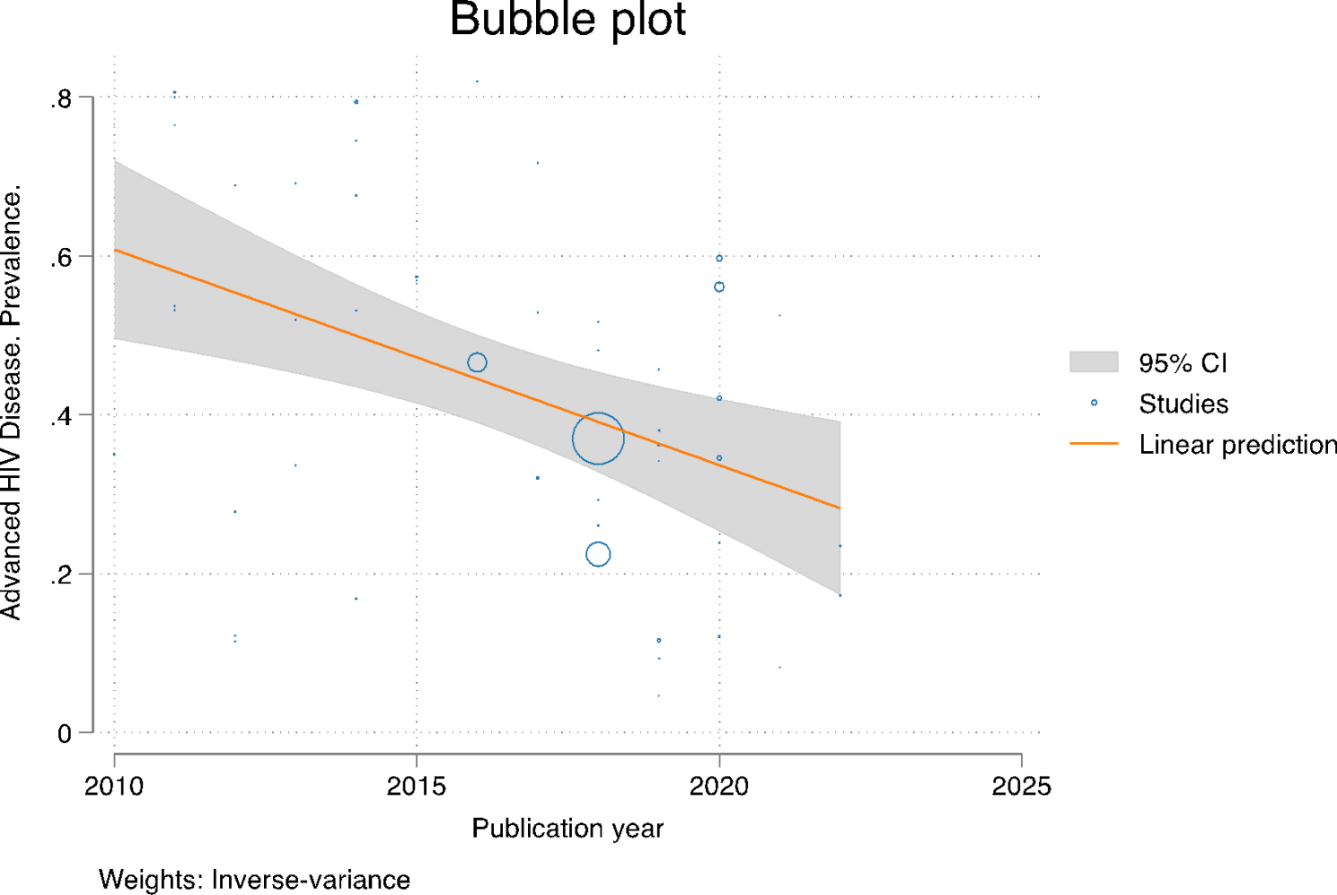
The time trend of advanced HIV disease from studies published during the period 2010–2022

In this study, we also attempted to assess the time trend of the prevalence of AHD among ART-naïve patients in South Africa from studies published from 2010 to 2022. The result indicated that there was a general linear trend of AHD declining in each successive year, (coefficient: -0.02, 95% CI -0.03 to 0.01), Fig. 6. Similarly, cumulative meta-analysis showed that the pooled prevalence of AHD among ART-naïve declined over time.

### Meta-regression

All meta-analyses in this review exhibited a substantial heterogeneity in pooled estimates, with subgroup and time trend analysis demonstrating potential associations with age, year of publication, study setting, and the AHD assessment method. In the adjusted meta-regression analysis, the period in which the selected studies were published was the only factor that remained associated with the prevalence of AHD (crude meta-regression coefficient: -0.02, 95% CI -0.03 to 0.01), meaning the prevalence of ADH among ART-naïve patients declined in each successive year by 2%, Table 2.

**Table 2:** Crude and adjusted meta-regression analysis of the prevalence of AHD among ART-naïve patients

	Crude Coefficient	95% CI	p-value	Adjusted Coefficient	95% CI	p-value
Publication year*	− 0.02	-0.04 to -0.01	**0.010**	-0.02	-0.03 to 0.01	**0.048**
Data collection (Cross-sectional vs. Other)	0.04	-0.07 to 0.17	0.450	-0.02	-0.14 to 0.10	0.756
Setting (Inpatient vs. Outpatient)	-0.23	-0.44 to -0.03	0.026	-0.18	-0.40 to 0.04	0.109
Methodological Quality Score*	-0.02	-0.07 to 0.01	0.224	-0.01	-0.05 to 0.04	0.788
Assessment method (WHO Stage vs. CD4 count)	0.16	0.01 to 0.32	**0.048**	0.10	-0.05 to 0.26	0.202

*These explanatory variables were treated as continuous in the meta-regression model and the remaining variables were treated as categorical variables.

### Publication bias

Conventional funnel plots suggested that there was no evidence of publication bias in the meta-analysis. Additionally, the results of Egger and Begg’s tests were in accordance with the funnel plots, which suggested no publication bias (Begg’s p-value = 0.483 and Egger’s p-value = 0.2055), Fig. 7.

**Fig. 7:**
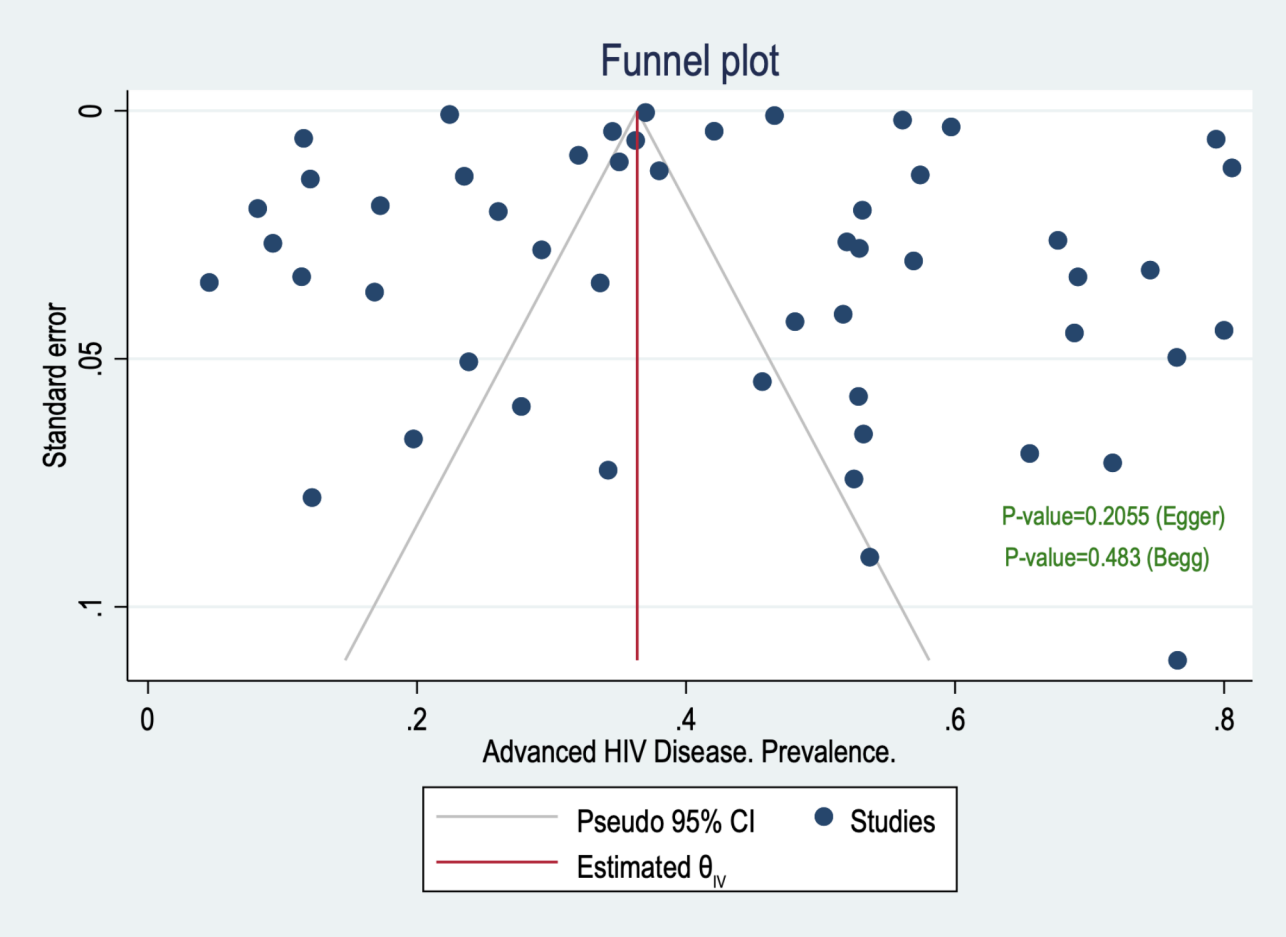
Publication bias prevalence estimate, from studies published during the period 2010–2022

Trim and fill method was also used to assess publication bias for the prevalence of AHD among ART-naïve patients. Under the random-effects model, the point estimate for the pooled prevalence of AHD was 43.4% (95% CI 40.1–46.8%). Using Trim and Fill, the imputed point estimate was marginally different from the pooled prevalence with 41.4% (95% CI 35.2–47.6%), the method suggests a total of 2 studies were missing from this review for the prevalence estimate.

## Discussion

This systematic review analysed 53 studies from 5 provinces in South Africa, involving 11,545,460 patients who received ART services between 2010 and 2022. The majority of the studies were cross-sectional analyses (24 studies), followed by longitudinal analysis of HIV programmatic data (13 studies), cross-sectional surveys (2 studies), prospective cohort studies (10 studies), and retrospective studies (4 studies). The overall prevalence of AHD among patients presenting to HIV services between 2010 and 2021 was 43.4%, however, there was substantial heterogeneity with this ranging between 4.5 and 83.1%. Due to the high heterogeneity between included studies, the pooled prevalence estimate should be interpreted with caution. Estimation of prediction intervals showed narrow range of expected prevalence of AHD among ART-naïve patients between 38.0% and 50.0% in a future, new systematic review and meta-analysis.

Subgroup analyses related to AHD diagnostic methods (CD4 versus WHO) and age did not explain the observed heterogeneity. Among the studies (n = 2) reporting on the presentation of ART-experienced patients, AHD was 58.6%. A time trend analysis revealed a decline of approximately 9.0% in the prevalence of AHD over the 10 years, with a prevalence of 52.4% in 2010 and 43.4% in 2022.

Since the start of the HIV programme, CD4 counts have been used to guide treatment decisions and identify those at risk for morbidity, mortality, and increased medical and psychosocial needs [5]. During the review period ART initiation guidelines were revised several times (patients were deemed eligible for ART if their CD4 count was < 200 cells/mm^3^ prior to 1 May 2010 or < 350 cells/mm^3^ after 1 May 2010 and 500 cells/mm^3^ between January 2015 and 31 August 2015) to expand form ART eligibility based on CD4 count and other co-morbidity, to all PLHIV becoming eligible for ART irrespective of CD4 count by 2016. As such, prior to and in 2010 the majority of PLHIV were at risk of starting ART with AHD whereas after 2010 this decreases due to guideline changes [5, 6].

Our study found that the proportion of patients presenting with AHD remains high but has decreased over time due to increased HIV testing and access to ART. Time trend analysis showed a 2% yearly decline in AHD among ART-naive patients. Our findings align with previous studies from both high-income and low- and middle-income settings [11, 14–16, 28, 29], which also reported either a high proportion or a modest decline in patients with AHD. Given the moderate decline in the proportion of AHD among ART-naïve patients, these results emphasize the importance of expanding HIV testing and timely linkage to care, to promote early ART initiation.

A meta-analysis data, which reported a mean CD4 count at ART initiation in 27 countries of 186 cells/mm^3^, suggest at least one in four ART patients in SSA initiated ART with advanced disease in 2015 and CD4 count at presentation to care and at ART initiation have not increased over the past decade [16]. When restricted analysis to studies conducted in South Africa, the meta-analysis found a statistically significant increase in CD4 counts at presentation but no change at ART initiation. To reduce the prevalence of AHD among ART-naïve patients in South Africa, continued attention to programmatic strategies facilitating earlier HIV testing and linkage to care are needed, including referral to primary health care and post-discharge support for adherence and retention, as well as implementing WHO-recommended universal ART eligibility (“treat-all policy”) guidelines for PLHIV [30, 31]. Furthermore, community awareness of AHD needs to be increased, as it is still limited [31].

Our study also found that 58.6% of patients with AHD were ART-experienced, this is a newly alarming trend observed in countries with long-standing HIV treatment programs and even in high ART coverage settings. Among ART-experienced, progress to AIDS either because they either interrupt treatment or because of treatment failure. The decline in the number of patients presenting for the first time with AHD could be offset by an increasing number of patients on long-term ART who have interrupted, stopped, or failed therapy [32–34]. Only two studies contributed to this outcome, therefore we cannot draw a firm conclusion using this finding. This evidence is consistent with findings from a multi-country cohort study conducted in 2 SSA countries evaluating characteristics and hospital outcomes of HIV-infected patients which showed that 65.1% of patients were admitted with AHD. Among inpatients with AHD, 71.7% were ART-experienced at admission with a median of 55.9 months on treatment (2). This means ongoing transmission due to ongoing viraemia among those who are out of care [36]. This supports a need for strategies to reduce gaps in HIV care and encourage return (re-engagement interventions such as welcome back services) before marked deterioration [37, 38], Given that more and more people who are in HIV programs are treatment experienced. This work supports the current reconceptualized non-linear HIV care cascade, which accounts for the cycling of patients in and out of care and the mobile populations served by ART programs in many SSA countries [12].

Furthermore, our subgroup analyses revealed a statistically significant difference in the prevalence of AHD among ART-naive patients between studies that used the WHO clinical staging system and those that used CD4 count criteria to identify patients with AHD, with 58.78% and 40.52% respectively. The higher prevalence among studies using the WHO clinical staging system should be interpreted with caution due to the poor accuracy associated with the WHO clinical staging system [39]. A systematic review and meta-analysis assessing the accuracy of WHO clinical staging system compared with CD4 count found that the WHO clinical stage 3 or 4 had a sensitivity of 60% and specificity of 73% for a CD4 threshold of ≤ 200 cells/mm3, and that sensitivity and specificity decreased as the CD4 count threshold increased [40]. Therefore, relying on the WHO clinical staging system alone risks missing a substantial number of PLHIV with severe immune suppression. Additionally, a multi-country study from Kenya, Malawi, Uganda, and Zimbabwe found that almost half of the people with CD4 count < 100 cells/mm3 were classified as having WHO clinical stage 1 or 2 disease [41].

The role of the CD4 count in HIV management is changing, with a shift away from using CD4 count to decide when to start ART or monitor treatment efficacy. Nevertheless, CD4 count remains the best measurement of patients’ immune and clinical status, the risk of opportunistic infections, and diagnosis decision-making, particularly for patients with AHD [30, 42].

Hence, identifying people with AHD who are eligible for elements of a package of care requires CD4 cell count testing. In addition, determining the immune status of PLHIV whose treatment is failing by virological criteria can help in guiding clinical management decisions (providing a package of care to reduce mortality and morbidity among people with AHD, which package includes screening, treatment, and/or prophylaxis for major opportunistic infections, rapid ART initiation and intensified adherence support interventions) [43].

Furthermore, the shift from monitoring CD4 cell count to monitoring viral load among individuals on ART has contributed to the de-prioritisation of testing CD4 cell count. Monitoring of viral load and CD4 cell count complement one another and should not be placed in competition. Monitoring VL supports viral suppression and triage into differentiated models of care, whereas CD4 cell count identifies people for whom an additional intervention is most urgently needed [44]. For these reasons, WHO recommends that HIV programs should retain the capacity to perform CD4 cell count at baseline and in the case of virological failure.

### Strengths and limitations

To the best of our knowledge, this is one of the first reviews to investigate the prevalence of AHD among ART-naïve and ART-experienced patients in South Africa. Of the 53 studies included in our meta-analysis, 13 were longitudinal analyses of HIV programmatic data, estimating the burden of AHD in real-world conditions at public health facilities, therefore maximizing generalizability. A further strength of this review is that we comprehensively searched for all eligible studies in all major databases and rigorously carried out the study following the PRISMA guidelines.

This review has several limitations that warrant discussion. Firstly, caution should be exercised when evaluating the current review results as there was substantial heterogeneity among included studies; this is not uncommon when evaluating systematic reviews of this nature. The impact of study quality on pooled prevalence was assessed by conducting meta-regression. Meta-regression demonstrated no evidence of data collection method or methodological quality score or assessment method or setting (inpatients versus outpatients).

A further limitation of this review is that gender-stratified data are not reported in most of the studies, making them unusable in the current meta-analysis. Although programs described patients as ART-naïve, many people who present to HIV service as ART-naïve patients may in fact be ART-experienced but have been out of care and are re-presenting as though they have never been in care before. Meta-analysis assumes that the data from contributing studies are independent of each other. However, there may be overlap in the patients summarized in the included studies, especially for those longitudinal studies using HIV program data, thus violating the assumption of data independence required for meta-analysis, introducing bias to the overall estimate, and that results would be biased towards the results of those patients included more than once.

### Implications for practice and future research

The current evidence suggests that the proportion of AHD among ART-naïve patients has declined over time (during the observed period) but remains consistently high. Therefore, CD4 count measurements remain important to identify people with AHD despite the introduction of the treat-all guidelines in 2016. These findings highlight the ongoing need for baseline CD4 count measurement to identify a large number of patients with AHD among ART-naïve patients, in order to provide differentiated models of care for this group of patients [5], guide prophylaxis against opportunistic infections (severe bacterial infections, TB and cryptococcal disease), and reduce mortality [41, 45–48]. Our findings also emphasize the importance of maintaining CD4 count testing capacity and infrastructure if donor funding for the CD4 measurement is reduced as global efforts prioritize the scale-up of VL monitoring in LMICs.

Capacity building and training for healthcare workers are also needed to quickly identify and timeously treat patients who are critically ill to save lives. This includes addressing health care workers’ attitude to be welcoming and understanding to patients re-engaging back into care.

Further research priorities should focus to assess the impact of universal test and treat guidelines on CD4 count testing at ART initiation among adults. Further research is needed on evaluating long-term outcomes among ART-naïve patients with advanced HIV disease pre- and post-universal test and treat guidelines under field conditions.

## Conclusion

Despite progress in making ART available to millions of PLHIV in South Africa, the burden AHD among ART-naive and ART-experienced patients remains consistently high. This is despite South Africa having a well-established ART program with excellent population-level coverage. Our findings highlight that CD4 cell count measurement remains an essential component of HIV care and is critically important in identifying individuals with low CD4 counts and enabling differentiated care focused on reducing the high morbidity and mortality in this vulnerable patient group. These results emphasize the importance of expanding HIV testing and timely linkage to care, to promote early ART initiation. There is a need to programmatically improve and decentralize screening for AHD at primary health clinics.

### Electronic supplementary material

Below is the link to the electronic supplementary material.

**Supplementary files: Appendix 1**: Search Strategy. **Appendix 2**: Risk of bias and quality of included studies. **Appendix 3**: **Supplementary figures. Figure 1** Prevalence of AHD among ART-naïve by study setting (hospital versus outpatients). **Figure 2**. Prevalence of AHD among ART-naïve patients by study design. **Figure 3**. Prevalence of AHD among ART-naïve patients by province. **Figure 4**. A sensitivity analysis of the prevalence of AHD among ART-naïve patients in South Africa when each indicated studies are removed at a time with its 957% confidence interval.

## Data Availability

Data will be available upon reasonable request of the corresponding.

## List of abbreviations

AHD: Advanced HIV Disease
AIDS: Acquired Immune Deficiency Syndrome
ART: Anti-Retroviral Therapy
CD4: Cluster of Differentiation 4
CrAg: Cryptococcal antigen
EPTB: Extra-pulmonary tuberculosis
GXP: GeneXpert
HIV: Human Immunodeficiency Virus
LMIC: Low- and middle-income countries
PLWHIV: People living with HIV
PRISMA: Preferred Reporting Items for Systematic Reviews and Meta-analyses
PROSPERO: Prospective Register of Systematic Reviews
SSA: Sub-Saharan Africa
TB: Tuberculosis
TB LAM: Tuberculosis lipoarabinomannan antigen
VL: Viral Load
WHO: Woold Health Organization
