# Supplementary material for: Prevalence and trends of advanced HIV disease among antiretroviral therapy-naïve and antiretroviral therapy-experienced patients in South Africa between 2010-2021: a systematic review and meta-analysis
Source: BMC Infect Dis. 2023 Aug 22;23:549. doi: 10.1186/s12879-023-08521-4 (PMC10464046; doi:10.1186/s12879-023-08521-4)
Supplement: Supplementary file 1 — Supplementary files: Appendix 1: Search Strategy. Appendix 2: Risk of bias and quality of included studies. Appendix 3: Supplementary figures. Figure 1 Prevalence of AHD among ART-naïve by study setting (hospital versus outpatients). Figure 2. Prevalence of AHD among ART-naïve patients by study design. Figure 3. Prevalence of AHD among ART-naïve patients by province. Figure 4. A sensitivity analysis of the prevalence of AHD among ART-naïve patients in South Africa when each indicated studies are removed at a time with its 957% confidence interval. [file 12879_2023_8521_MOESM1_ESM.docx]

**Supplementary files**

**Appendix 1: Search Strategy**

1. **PubMed**

|  |  | Records |
| --- | --- | --- |
| #1 | Search: ((HIV OR hiv-1 OR hiv-2* OR hiv1 OR hiv2 OR hiv infect* OR human immunodeficiency virus OR human immune deficiency virus OR human immuno-deficiency virus OR human immune-deficiency virus OR ((human immun*) AND (deficiency virus)) OR acquired immunodeficiency syndromes OR acquired immune deficiency syndrome OR acquired immuno-deficiency syndrome OR acquired immune-deficiency syndrome OR ((acquired immun*) AND (deficiency syndrome)) OR HIV/AIDS)) | 102,054 |
| #2 | Search: ((HIV infections [MeSH] OR HIV [MeSH])) | 293,403 |
| #3 | (#1 OR #2) | 438,271 |
| #4 | Search: (“AIDS-defining disease”  OR  “AIDS-defining illness”  OR  “AIDS-related disease”  OR  “AIDS-related illness”  OR  “CD4 cell count less than 200”  OR  “CD4 cell count below 200”  OR  “CD4 200”  OR  “CD4 cell count 200”  OR  “CD4 T cell count 200”  OR  “CD4 T lymphocytes less than 200”  OR  “CD4 T lymphocytes below 200”  OR  “CD4 T lymphocytes 200”  OR  “HIV test*” OR WHO stage 3 OR WHO stage 3 ) | 521,550 |
| #5 | Search: (#3 AND #4) | 22,930 |
| #6 | Search: ((((((((("Prevalence"[Mesh] OR "Epidemiology"[Mesh] OR "Cross-Sectional Studies"[Mesh])) OR  Prevalence) OR Epidemiology) OR Cross-Sectional Studies) OR "Longitudinal Studies"[Mesh]) OR  "Cohort Studies"[Mesh]) OR Cohort Studies) OR Longitudinal Studies)) | 311,478 |
| #7 | Search: (#6AND #5) | 5,636 |
| #8 | Search: ("Africa"[MeSH] OR Africa*[tw] OR "South Africa"[tw] | 936,251 |
| #9 | Search: (#8 AND #7) | 1,658 |

1. **Scopus**

|  |  | Records |
| --- | --- | --- |
| #1 | TITLE-ABS-KEY (“human AND immunodeficiency AND virus” OR hiv OR “hiv AND infection” OR “aids AND virus” OR “acquired AND immune AND deficiency AND syndrome AND virus AND “ ) | 76,896 |
| #2 | TITLE-ABS-KEY ( “AIDS-defining disease”  OR  “AIDS-defining illness”  OR  “AIDS-related disease”  OR  “AIDS-related illness”  OR  “CD4 cell count less than 200”  OR  “CD4 cell count below 200”  OR  “CD4 200”  OR  “CD4 cell count 200”  OR  “CD4 T cell count 200”  OR  “CD4 T lymphocytes less than 200”  OR  “CD4 T lymphocytes below 200”  OR  “CD4 T lymphocytes 200”  OR  “HIV test*” OR WHO stage 3 OR WHO stage 3 ) | 20,229 |
| #3 | #1 OR #2 | 94,256 |
| #4 | TITLE-ABS-KEY (“advanced HIV disease” OR late OR “late diagnosis” OR “delayed diagnosis” OR “late present*” OR “late HIV diagnosis” OR “late test*”) | 2,038,593 |
| #5 | TITLE-ABS-KEY ((((((((("Prevalence"[Mesh] OR "Epidemiology"[Mesh] OR "Cross-Sectional Studies"[Mesh])) OR  Prevalence) OR Epidemiology) OR Cross-Sectional Studies) OR "Longitudinal Studies"[Mesh]) OR  "Cohort Studies"[Mesh]) OR Cohort Studies) OR Longitudinal Studies)) | 4,159,113 |
| #6 | #4 AND #5 | 250,515 |
| #7 | #3 AND #6 | 1372 |
| #8 | TITLE-ABS-KEY (africa* OR south africa) | 151,344 |
| #9 | #7 AND #8 | 110 |
| #10 | ( TITLE-ABS-KEY ( africa*  OR  South Africa) )  AND  ( ( ( TITLE-ABS-KEY ( “human  AND immunodeficiency  AND virus”  OR  hiv  OR  “hiv  AND infection”  OR  “aids  AND virus”  OR  “acquired  AND immune  AND deficiency  AND syndrome  AND virus  AND “ ) )  OR  ( TITLE-ABS-KEY ( "AIDS-defining disease"  OR  "AIDS-defining illness"  OR  "AIDS-related disease"  OR  "AIDS-related illness"  OR  "CD4 cell count less than 200"  OR  "CD4 cell count below 200"  OR  "CD4 200"  OR  "CD4 cell count 200"  OR  "CD4 T cell count 200"  OR  "CD4 T lymphocytes less than 200"  OR  "CD4 T lymphocytes below 200"  OR  "CD4 T lymphocytes 200"  OR  "HIV test*" ) ) )  AND  ( ( TITLE-ABS-KEY ( "advanced HIV disease"  OR  late  OR  "late diagnosis"  OR  "delayed diagnosis"  OR  "late present*"  OR  "late HIV diagnosis"  OR  "late test*" ) )  AND  ( TITLE-ABS-KEY ( burden  OR  prevalen*  OR  occurre*  OR  cross-sectional ) ) ) )  AND  ( LIMIT-TO ( PUBYEAR ,  2021 )  OR  LIMIT-TO ( PUBYEAR ,  2020 )  OR  LIMIT-TO ( PUBYEAR ,  2019 )  OR  LIMIT-TO ( PUBYEAR ,  2018 )  OR  LIMIT-TO ( PUBYEAR ,  2017 )  OR  LIMIT-TO ( PUBYEAR ,  2016 )  OR  LIMIT-TO ( PUBYEAR ,  2015 )  OR  LIMIT-TO ( PUBYEAR ,  2014 )  OR  LIMIT-TO ( PUBYEAR ,  2013 )  OR  LIMIT-TO ( PUBYEAR ,  2012 )  OR  LIMIT-TO ( PUBYEAR ,  2011 )  OR  LIMIT-TO ( PUBYEAR ,  2010 )  OR  LIMIT-TO ( PUBYEAR ,  2009 )  OR  LIMIT-TO ( PUBYEAR ,  2008 )  OR  LIMIT-TO ( PUBYEAR ,  2007 )  OR  LIMIT-TO ( PUBYEAR ,  2006 )  OR  LIMIT-TO ( PUBYEAR ,  2005 )  OR  LIMIT-TO ( PUBYEAR ,  2004 )  OR  LIMIT-TO ( PUBYEAR ,  2003 )  OR  LIMIT-TO ( PUBYEAR ,  2002 )  OR  LIMIT-TO ( PUBYEAR ,  2001 ) ) | 77 |

1. **Websicence**

|  |  | records |
| --- | --- | --- |
| #1 | **TOPIC:** (“Human immunodeficiency virus” OR HIV OR “HIV Infection” OR “AIDS Virus” or “Acquired Immune Deficiency Syndrome Virus “) | 418,054 |
| #2 | \|  \| **TOPIC:** (“AIDS-defining disease” OR” AIDS-defining illness” OR “AIDS-related disease” OR “AIDS-related illness” OR “CD4 cell count less than 200” OR “CD4 cell count below 200” OR “CD4 < 200” OR “CD4 cell count < 200” OR “CD4 T cell count < 200” OR “CD4 T lymphocytes less than 200” OR “CD4 T lymphocytes below 200” OR “CD4 T lymphocytes < 200” OR “HIV test*”) \| \| --- \| --- \| | 15,300 |
| #3 | #2 OR #1 | 418,133 |
| #4 | **TOPIC:** (“advanced HIV disease” OR Late OR “late diagnosis” OR “delayed diagnosis” OR “late present*” OR “late HIV diagnosis” OR WHO stage 3 OR WHO stage 3 OR “late test*”) | 1,432,688 |
| #5 | \|  \| #4 AND #3 \| \| --- \| --- \| | 14,466 |
| #6 | TS= (Africa* OR “South Africa”) | 1,254,407 |
| #7 | #6 AND #5 | 2,748 |
| #8 | #6 AND #5 **Refined by:** **PUBLICATION YEARS:** (2021 OR 2013 OR 2005 OR 2020 OR 2012 OR 2004 OR 2019 OR 2011 OR 2003 OR 2018 OR 2010 OR 2002 OR 2017 OR 2009 OR 2001 OR 2016 OR 2008 OR 2000 OR 2015 OR 2007 OR 2014 OR 2006 )) | 2,562 |
| #9 | #6 AND #5 **Refined by:** **PUBLICATION YEARS:** (2021 OR 2013 OR 2005 OR 2020 OR 2012 OR 2004 OR 2019 OR 2011 OR 2003 OR 2018 OR 2010 OR 2002 OR 2017 OR 2009 OR 2001 OR 2016 OR 2008 OR 2000 OR 2015 OR 2007 OR 2014 OR 2006) AND [excluding] **WEB OF SCIENCE INDEX:** (WOS.ISTP OR WOS.AHCI OR WOS.SSCI OR WOS.BSCI OR WOS.BHCI OR WOS.ESCI OR WOS.ISSHP) | 1,481 |

1. **Scielo**

|  |  | records |
| --- | --- | --- |
| #1 | **TOPIC:** (“Human immunodeficiency virus” OR HIV OR “HIV Infection” OR “AIDS Virus” or “Acquired Immune Deficiency Syndrome Virus “) | **562,731** |
| #2 | \|  \| **TOPIC:**  (“AIDS-defining disease” OR ”AIDS-defining illness” OR “AIDS-related disease” OR “AIDS-related illness” OR “CD4 cell count less than 200” OR “CD4 cell count below 200” OR “CD4 < 200” OR “CD4 cell count < 200” OR “CD4 T cell count < 200” OR “CD4 T lymphocytes less than 200” OR “CD4 T lymphocytes below 200” OR “CD4 T lymphocytes < 200” OR “HIV test*” OR “WHO stage 3” OR “WHO stage 3” ) \| \| --- \| --- \| | 21,066 |
| #3 | #2 OR #1 | 562,840 |
| #4 | **TOPIC:** (“advanced HIV disease” OR Late OR “late diagnosis” OR “delayed diagnosis” OR “late present*” OR “late HIV diagnosis” OR “late test*”) | 1,975,161 |
| #5 | \|  \| #4 AND #3 \| \| --- \| --- \| | 20,360 |
| #6 | TS= (Africa* OR South Africa) | 1,712,142 |
| #7 | #6 AND #5 | 4,148 |
| #8 | #6 AND #5 **Refined by:** **Refined by:** **PUBLICATION YEARS:** (2021 OR 2011 OR 2001 OR 2020 OR 2010 OR 2000 OR 2019 OR 2009 OR 2018 OR 2008 OR 2017 OR 2007 OR 2016 OR 2006 OR 2015 OR 2005 OR 2014 OR 2004 OR 2013 OR 2003 OR 2012 OR 2002) | 3,549 |
| #9 | #6 AND #5 **Refined by:** **PUBLICATION YEARS:** (2021 OR 2011 OR 2001 OR 2020 OR 2010 OR 2000 OR 2019 OR 2009 OR 2018 OR 2008 OR 2017 OR 2007 OR 2016 OR 2006 OR 2015 OR 2005 OR 2014 OR 2004 OR 2013 OR 2003 OR 2012 OR 2002) AND [excluding] **Databases:** (WOS) | 337 |

1. **AfricaWide**

|  |  | records |
| --- | --- | --- |
| #1 | (“Human immunodeficiency virus” OR HIV OR “HIV Infection” OR “AIDS Virus” or “Acquired Immune Deficiency Syndrome Virus “) | 119,086 |
| #2 | \|  \|  \| \| --- \| --- \|   (“AIDS-defining disease” OR ”AIDS-defining illness” OR “AIDS-related disease” OR “AIDS-related illness” OR “CD4 cell count less than 200” OR “CD4 cell count below 200” OR “CD4 < 200” OR “CD4 cell count < 200” OR “CD4 T cell count < 200” OR “CD4 T lymphocytes less than 200” OR “CD4 T lymphocytes below 200” OR “CD4 T lymphocytes < 200” OR “HIV test*” OR “WHO stage 3” OR “WHO stage 3”) | 7,394 |
| #3 | #1 OR #2 | 7394 |
| #4 | (“advanced HIV disease” OR Late OR “late diagnosis” OR “delayed diagnosis” OR “late present*” OR “late HIV diagnosis” OR “late test*”) | 51,170 |
| #5 | \|  \|  \| \| --- \| --- \|   #3 AND #4 | 322 |
| #6 | (Africa* OR "South Africa”) **Refined by:** **PUBLICATION YEARS:** (2021 OR 2011 OR 2001 OR 2020 OR 2010 OR 2000 OR 2019 OR 2009 OR 2018 OR 2008 OR 2017 OR 2007 OR 2016 OR 2006 OR 2015 OR 2005 OR 2014 OR 2004 OR 2013 OR 2003 OR 2012 OR 2002) | 3,758,884 |
| #7 | #5 AND #6 | 261 |

1. **CINAHL**

|  |  | records |
| --- | --- | --- |
| #1 | (“Human immunodeficiency virus” OR HIV OR “HIV Infection” OR “AIDS Virus” or “Acquired Immune Deficiency Syndrome Virus “) | 120,236 |
| #2 | \|  \|  \| \| --- \| --- \|   (“AIDS-defining disease” OR ”AIDS-defining illness” OR “AIDS-related disease” OR “AIDS-related illness” OR “CD4 cell count less than 200” OR “CD4 cell count below 200” OR “CD4 < 200” OR “CD4 cell count < 200” OR “CD4 T cell count < 200” OR “CD4 T lymphocytes less than 200” OR “CD4 T lymphocytes below 200” OR “CD4 T lymphocytes < 200” OR “HIV test*” OR “WHO stage 3” OR “WHO stage 3”) | 7,730 |
| #3 | #1 OR #2 | 7,730 |
| #4 | (“advanced HIV disease” OR Late OR “late diagnosis” OR “delayed diagnosis” OR “late present*” OR “late HIV diagnosis” OR “late test*”) | 73,842 |
| #5 | \|  \|  \| \| --- \| --- \|   #3 AND #4 | 342 |
| #6 | (Africa* OR "South Africa") | 155,105 |
| #7 | #5 AND #6 | 89 |
| #8 | (Africa* OR "South Africa") **Refined by:** **PUBLICATION YEARS:** (2021 OR 2011 OR 2001 OR 2020 OR 2010 OR 2000 OR 2019 OR 2009 OR 2018 OR 2008 OR 2017 OR 2007 OR 2016 OR 2006 OR 2015 OR 2005 OR 2014 OR 2004 OR 2013 OR 2003 OR 2012 OR 2002) | 88 |

**Appendix 2: Risk of bias and quality of included studies**

| **Studies** | **1. Was the target population representative of the population in relation to relevant studies?** | **2. Was the sampling frame a true or close representation of the target population** | **3. Was some form of random selection used to select the sample, OR was a census undertaken?** | **4. Was the likelihood of non-response bias minimal in the study?** | **5. Were data collected directly from the subjects (as opposed to a proxy)?** | **6. Was an acceptable case definition used in the study?** | **7. Was the study instrument that measured the parameter of interest shown to have validity and reliability?** | **8. Was the same mode of data collection used for all subjects?** | **9. Was the length of the shortest prevalence period for the parameter of interest appropriate?** | **10. Were the numerator(s) for the parameter of interest appropriate?** | **11. Summary score on the overall risk of study bias** | **Risk of bias** |
| --- | --- | --- | --- | --- | --- | --- | --- | --- | --- | --- | --- | --- |
| Adeniyi 2018 | √ | √ | √ | NS | √ | √ | NS | √ | √ | √ | 8 | Moderate |
| Bock 2018 | √ | √ | √ | NS | √ | √ | NS | √ | √ | √ | 8 | Moderate |
| Boulle 2014 | √ | NS | NS | √ | √ | √ | NS | √ | √ | √ | 7 | Moderate |
| Carmona 2018 | √ | NS | NS | √ | √ | √ | NS | √ | √ | √ | 7 | Moderate |
| Cassidy 2022 | √ | √ | √ | NS | √ | √ | √ | √ | √ | √ | 9 | Low |
| Chihana 2019 | √ | √ | √ | √ | √ | √ | √ | √ | √ | √ | 10 | Low |
| Cholera 2017 | √ | √ | √ | √ | √ | √ | √ | √ | √ | √ | 10 | Low |
| Clouse 2013 | √ | √ | √ | √ | √ | √ | √ | √ | √ | √ | 10 | Low |
| Conan 2019 | √ | √ | √ | √ | √ | √ | √ | √ | √ | √ | 10 | Low |
| Dorward 2020 | √ | √ | NS | NS | √ | √ | √ | √ | √ | √ | 7 | Moderate |
| DrainPaul 2013 | √ | NS | NS | √ | √ | √ | √ | √ | √ | √ | 8 | Moderate |
| Dramowski 2011 | √ | NS | NS | NS | NS | √ | √ | NS | √ | √ | 5 | High |
| Feucht 2016 | √ | √ | √ | √ | √ | √ | √ | √ | √ | √ | 8 | Moderate |
| Fomundam 2017 | √ | √ | NS | NS | √ | √ | √ | √ | √ | √ | 8 | Moderate |
| Glencross 2020 | √ | √ | NS | NS | √ | √ | √ | √ | √ | √ | 8 | Moderate |
| Haas 2020 | √ | √ | √ | √ | √ | √ | √ | √ | √ | √ | 10 | Low |
| Haddow 2012 | √ | √ | NS | NS | √ | √ | √ | √ | √ | √ | 8 | Moderate |
| Hunt 2017 | √ | NS | NS | NS | √ | √ | √ | √ | √ | √ | 7 | Moderate |
| Kamkuemah 2015 | √ | √ | √ | NS | √ | √ | NS | √ | √ | √ | 8 | Moderate |
| Kranzer 2012 | √ | NS | NS | √ | √ | √ | NS | √ | √ | √ | 7 | Moderate |
| Larsen 2019 | √ | √ | √ | √ | √ | √ | √ | √ | √ | √ | 10 | Low |
| Larson 2010 | √ | NS | NS | √ | √ | √ | NS | √ | √ | √ | 7 | Moderate |
| Lawn 2011 | √ | √ | √ | √ | √ | √ | √ | √ | √ | √ | 10 | Low |
| Lewis 2021 | √ | √ | √ | √ | √ | √ | √ | √ | √ | √ | 10 | Low |
| Lilian 2019 | √ | √ | √ | √ | √ | √ | √ | √ | √ | √ | 10 | Low |
| Lurie 2014 | √ | √ | √ | √ | √ | √ | √ | √ | √ | √ | 10 | Low |
| Maduna 2015 | √ | √ | NS | NS | √ | √ | √ | √ | √ | √ | 7 | Moderate |
| Magidson 2019 | √ | NS | NS | √ | √ | √ | √ | √ | √ | √ | 8 | Moderate |
| Manicklal 2014 | √ | NS | NS | NS | NS | √ | √ | NS | √ | √ | 5 | High |
| Maskew 2011 | √ | √ | √ | √ | √ | √ | √ | √ | √ | √ | 8 | Moderate |
| Meintjes 2015 | √ | √ | NS | NS | √ | √ | √ | √ | √ | √ | 8 | Moderate |
| Mnyani 2017 | √ | √ | NS | NS | √ | √ | √ | √ | √ | √ | 8 | Moderate |
| Naidoo 2014 | √ | √ | √ | √ | √ | √ | √ | √ | √ | √ | 10 | Low |
| Ndlovu 2014 | √ | √ | NS | NS | √ | √ | √ | √ | √ | √ | 8 | Moderate |
| Nglazi 2012 | √ | NS | NS | NS | √ | √ | √ | √ | √ | √ | 7 | Moderate |
| Nir 2021 | √ | √ | √ | NS | √ | √ | √ | √ | √ | √ | 9 | Low |
| Nyakato 2022 | √ | √ | √ | √ | √ | √ | √ | √ | √ | √ | 10 | Low |
| Oni 2011 | √ | √ | √ | √ | √ | √ | √ | √ | √ | √ | 10 | Low |
| Osler 2018 | √ | √ | √ | √ | √ | √ | √ | √ | √ | √ | 10 | Low |
| Otwombe 2013 | √ | √ | √ | √ | √ | √ | √ | √ | √ | √ | 10 | Low |
| Patel 2010 | √ | √ | NS | NS | √ | √ | √ | √ | √ | √ | 7 | Moderate |
| Patten 2020 | √ | NS | NS | √ | √ | √ | √ | √ | √ | √ | 8 | Moderate |
| Peter 2012 | √ | √ | NS | NS | √ | √ | √ | √ | √ | √ | 8 | Moderate |
| Rane 2018 | √ | √ | NS | NS | √ | √ | √ | √ | √ | √ | 8 | Moderate |
| Rossouw 2015 | √ | √ | √ | NS | √ | √ | √ | √ | √ | √ | 9 | Low |
| Shigayeva 2019 | √ | √ | √ | √ | √ | √ | √ | √ | √ | √ | 10 | Low |
| Sogbanmu 2019 | √ | √ | √ | √ | √ | √ | √ | √ | √ | √ | 10 | Low |
| Tendesayi 2016 | √ | √ | √ | √ | √ | √ | √ | √ | √ | √ | 10 | Low |
| Theron 2011 | √ | √ | √ | √ | √ | √ | √ | √ | √ | √ | 10 | Low |
| VanRie 2011 | √ | √ | NS | NS | √ | √ | √ | √ | √ | √ | 7 | Moderate |
| vanSchalkwyk 2020 | √ | NS | NS | √ | √ | √ | √ | √ | √ | √ | 8 | Moderate |
| Venter 2018 | √ | NS | NS | NS | NS | √ | √ | NS | √ | √ | 5 | High |
| Zaniewski 2020 | √ | √ | √ | √ | √ | √ | √ | √ | √ | √ | 8 | Moderate |

| **Risk of bias** | **Low if >8** | **Moderate if 6-8** | **High if ≤5** |
| --- | --- | --- | --- |
| Yes | √ |  |  |
| NS | Not Stated |  |  |

Risk of bias using the tool by Hoy et al, adapted by warfalli et al. High risk, and unclear scored as zero “0”, while low risk was scored as one “0” for each item. Total score out of 10 where depicts low overall risk and 0 high overall risk. See appendix 2 for more details.

| **Section and Topic** | **Item #** | **Checklist item** | **Location where item is reported** |
| --- | --- | --- | --- |
| **TITLE** | | |  |
| Title | 1 | Identify the report as a systematic review. | 1-3 |
| **ABSTRACT** | | |  |
| Abstract | 2 | See the PRISMA 2020 for Abstracts checklist. | 24-50 |
| **INTRODUCTION** | | |  |
| Rationale | 3 | Describe the rationale for the review in the context of existing knowledge. | 51-96 |
| Objectives | 4 | Provide an explicit statement of the objective(s) or question(s) the review addresses. | 92-96 |
| **METHODS** | | |  |
| Eligibility criteria | 5 | Specify the inclusion and exclusion criteria for the review and how studies were grouped for the syntheses. | 117-133 |
| Information sources | 6 | Specify all databases, registers, websites, organisations, reference lists and other sources searched or consulted to identify studies. Specify the date when each source was last searched or consulted. | 135-143 |
| Search strategy | 7 | Present the full search strategies for all databases, registers and websites, including any filters and limits used. | 143-146 |
| Selection process | 8 | Specify the methods used to decide whether a study met the inclusion criteria of the review, including how many reviewers screened each record and each report retrieved, whether they worked independently, and if applicable, details of automation tools used in the process. | 147-156 |
| Data collection process | 9 | Specify the methods used to collect data from reports, including how many reviewers collected data from each report, whether they worked independently, any processes for obtaining or confirming data from study investigators, and if applicable, details of automation tools used in the process. | 155-162 |
| Data items | 10a | List and define all outcomes for which data were sought. Specify whether all results that were compatible with each outcome domain in each study were sought (e.g. for all measures, time points, analyses), and if not, the methods used to decide which results to collect. | 157-160 |
|  | 10b | List and define all other variables for which data were sought (e.g. participant and intervention characteristics, funding sources). Describe any assumptions made about any missing or unclear information. | 160-162 |
| Study risk of bias assessment | 11 | Specify the methods used to assess risk of bias in the included studies, including details of the tool(s) used, how many reviewers assessed each study and whether they worked independently, and if applicable, details of automation tools used in the process. | 163-175 |
| Effect measures | 12 | Specify for each outcome the effect measure(s) (e.g. risk ratio, mean difference) used in the synthesis or presentation of results. | 177-183 |
| Synthesis methods | 13a | Describe the processes used to decide which studies were eligible for each synthesis (e.g. tabulating the study intervention characteristics and comparing against the planned groups for each synthesis (item #5)). | NA |
|  | 13b | Describe any methods required to prepare the data for presentation or synthesis, such as handling of missing summary statistics, or data conversions. | 178-179 |
|  | 13c | Describe any methods used to tabulate or visually display results of individual studies and syntheses. | 179-183 |
|  | 13d | Describe any methods used to synthesize results and provide a rationale for the choice(s). If meta-analysis was performed, describe the model(s), method(s) to identify the presence and extent of statistical heterogeneity, and software package(s) used. | 184-192 |
|  | 13e | Describe any methods used to explore possible causes of heterogeneity among study results (e.g. subgroup analysis, meta-regression). | 179-182 |
|  | 13f | Describe any sensitivity analyses conducted to assess robustness of the synthesized results. | 52-59 |
| Reporting bias assessment | 14 | Describe any methods used to assess risk of bias due to missing results in a synthesis (arising from reporting biases). | NA |
| Certainty assessment | 15 | Describe any methods used to assess certainty (or confidence) in the body of evidence for an outcome. | NA |
| **RESULTS** | | |  |
| Study selection | 16a | Describe the results of the search and selection process, from the number of records identified in the search to the number of studies included in the review, ideally using a flow diagram. | 200-203 |
|  | 16b | Cite studies that might appear to meet the inclusion criteria, but which were excluded, and explain why they were excluded. | 203-204 |
| Study characteristics | 17 | Cite each included study and present its characteristics. | 207-213 |
| Risk of bias in studies | 18 | Present assessments of risk of bias for each included study. | 1-4 |
| Results of individual studies | 19 | For all outcomes, present, for each study: (a) summary statistics for each group (where appropriate) and (b) an effect estimate and its precision (e.g. confidence/credible interval), ideally using structured tables or plots. | 216 |
| Results of syntheses | 20a | For each synthesis, briefly summarise the characteristics and risk of bias among contributing studies. | 1-4 |
|  | 20b | Present results of all statistical syntheses conducted. If meta-analysis was done, present for each the summary estimate and its precision (e.g. confidence/credible interval) and measures of statistical heterogeneity. If comparing groups, describe the direction of the effect. | 5-11 |
|  | 20c | Present results of all investigations of possible causes of heterogeneity among study results. | 9-10 |
|  | 20d | Present results of all sensitivity analyses conducted to assess the robustness of the synthesized results. | 52-59 |
| Reporting biases | 21 | Present assessments of risk of bias due to missing results (arising from reporting biases) for each synthesis assessed. | NA |
| Certainty of evidence | 22 | Present assessments of certainty (or confidence) in the body of evidence for each outcome assessed. | NA |
| **DISCUSSION** | | |  |
| Discussion | 23a | Provide a general interpretation of the results in the context of other evidence. | 130-140 |
|  | 23b | Discuss any limitations of the evidence included in the review. | 236-242 |
|  | 23c | Discuss any limitations of the review processes used. | None |
|  | 23d | Discuss implications of the results for practice, policy, and future research. | 244-278 |
| **OTHER INFORMATION** | | |  |
| Registration and protocol | 24a | Provide registration information for the review, including register name and registration number, or state that the review was not registered. | 100-102 |
|  | 24b | Indicate where the review protocol can be accessed, or state that a protocol was not prepared. | 102 |
|  | 24c | Describe and explain any amendments to information provided at registration or in the protocol. |  |
| Support | 25 | Describe sources of financial or non-financial support for the review, and the role of the funders or sponsors in the review. | Declaration |
| Competing interests | 26 | Declare any competing interests of review authors. | Declaration |
| Availability of data, code and other materials | 27 | Report which of the following are publicly available and where they can be found: template data collection forms; data extracted from included studies; data used for all analyses; analytic code; any other materials used in the review. | Declaration |

**Appendix 3 : Supplementary figures**


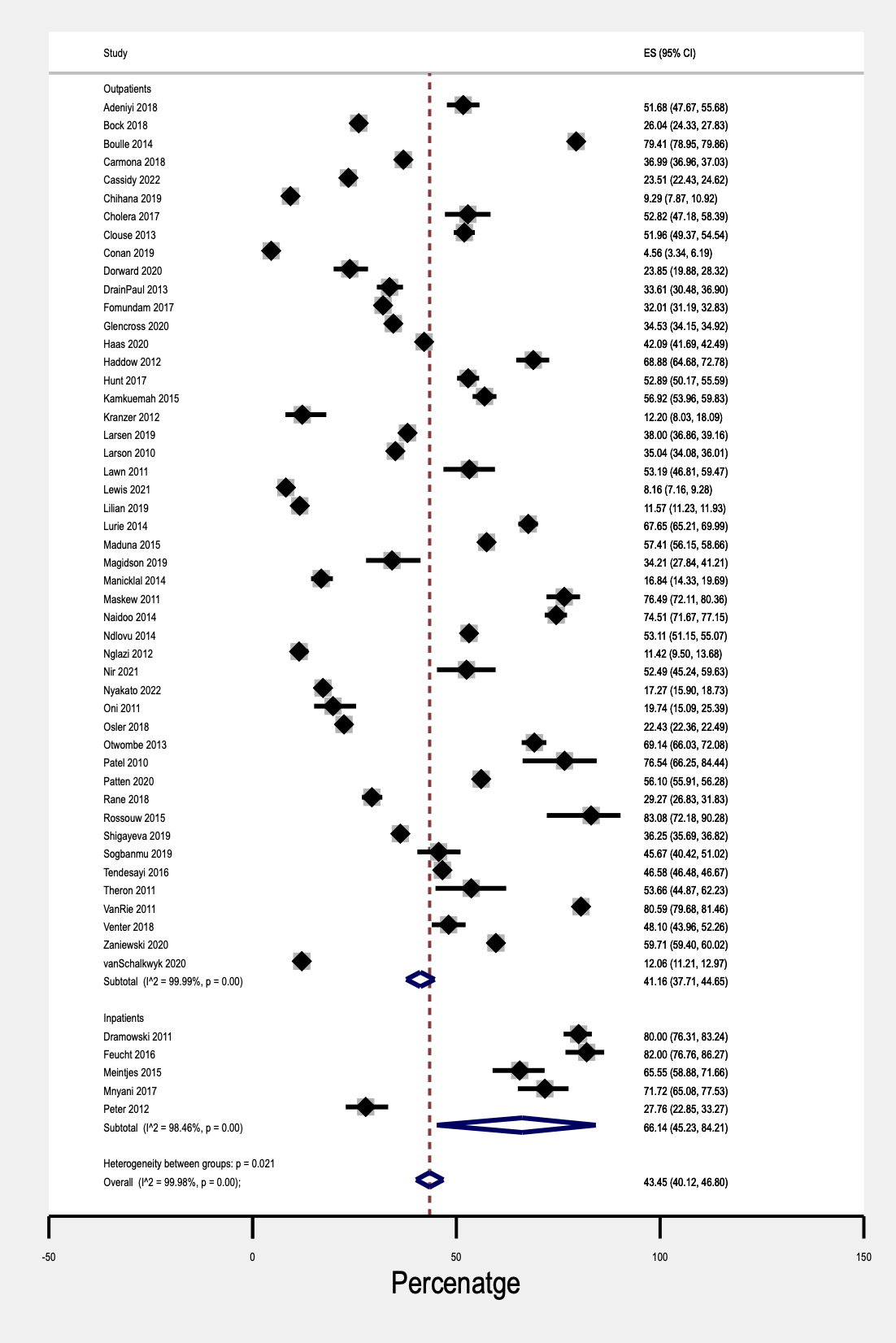


**Figure 1 Prevalence of AHD among ART-naïve by study setting (hospital versus outpatients)**


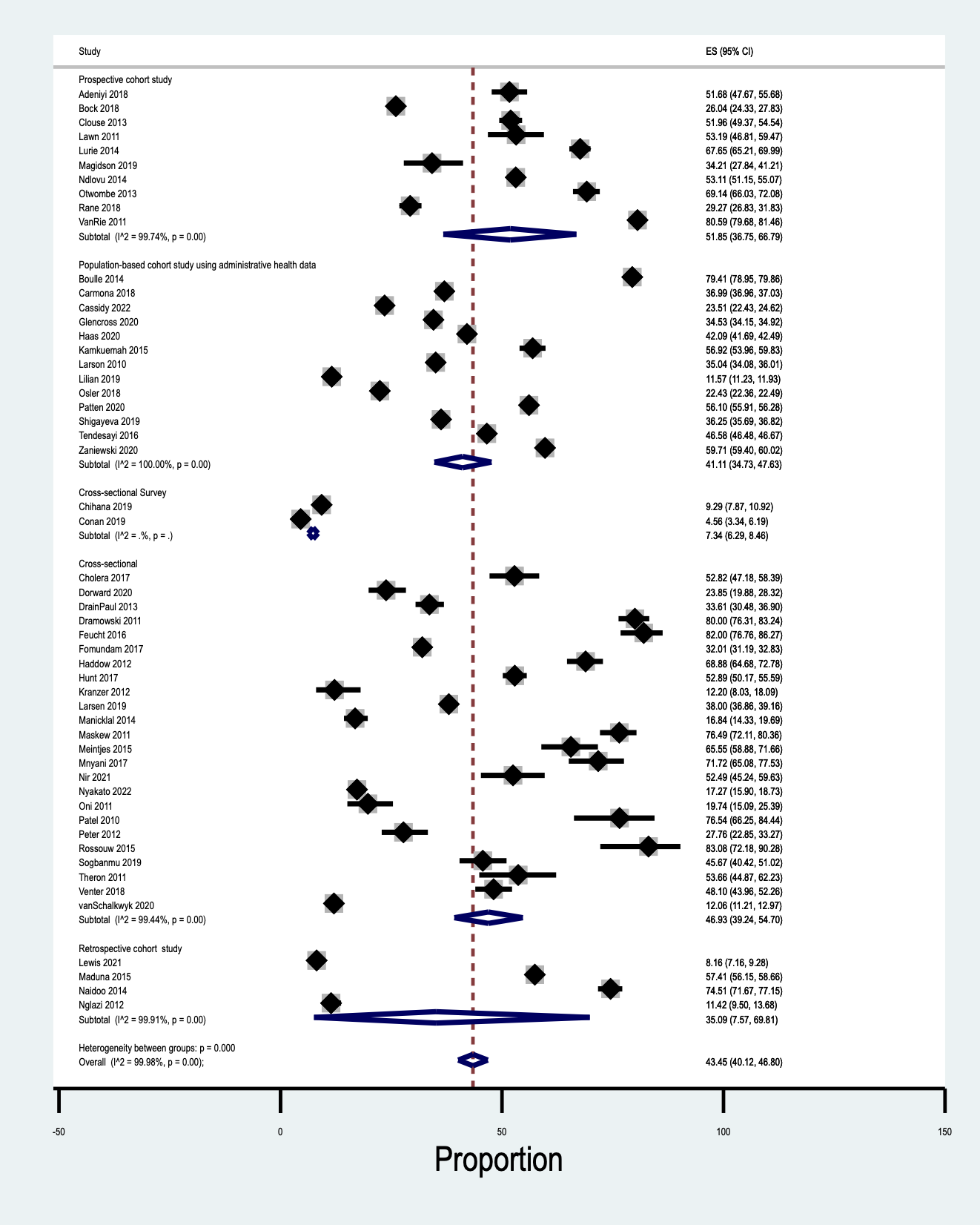


**Figure 2. Prevalence of AHD among ART-naïve patients by study design**

**Figure 3. Prevalence of AHD among ART-naïve patients by province**

**
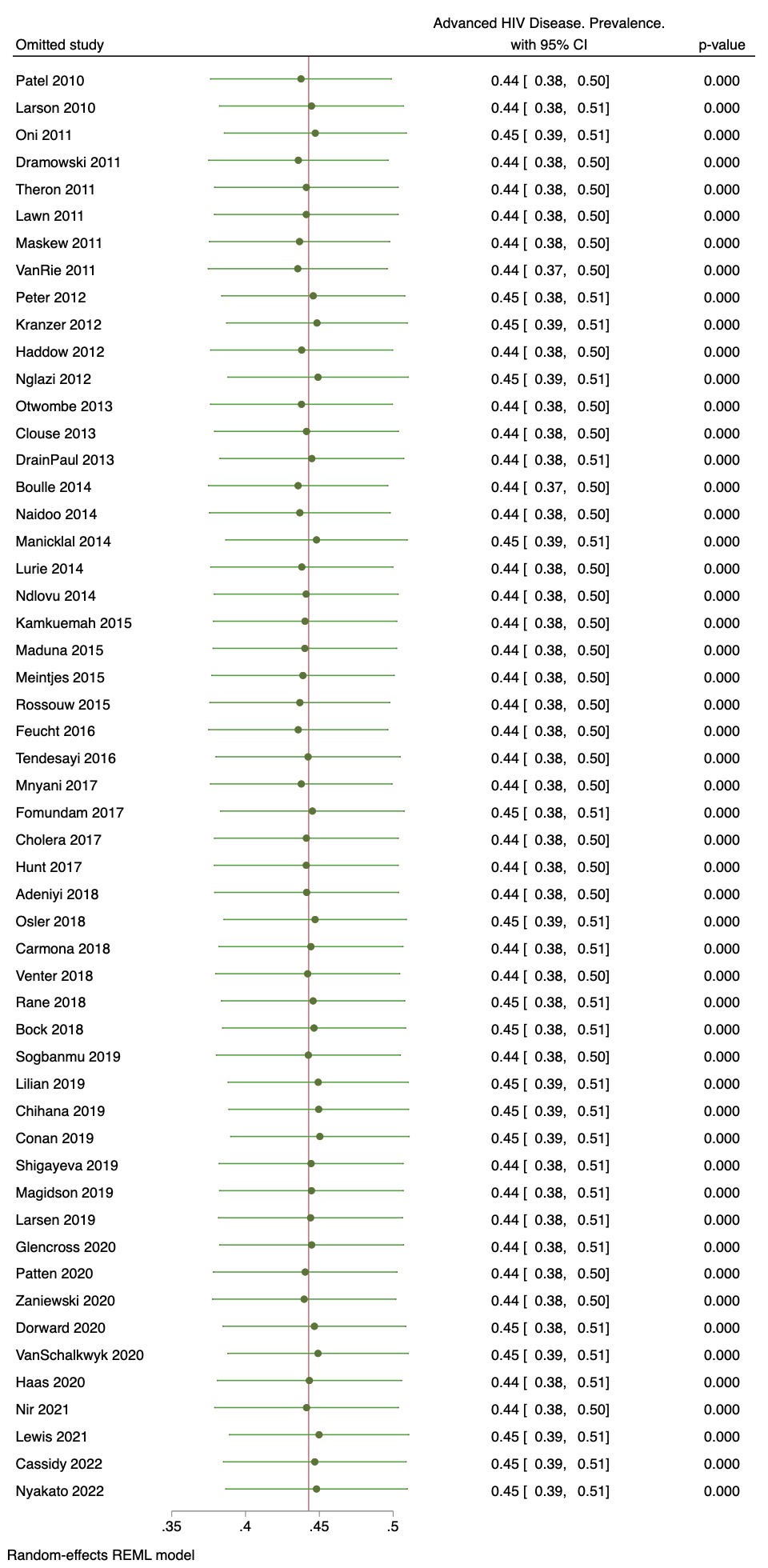
**

**Figure 4. A sensitivity analysis of the prevalence of AHD among ART-naïve patients in South Africa when each indicated studies are removed at a time with its 95% confidence interval**.
